# Evaluation of repair activity by quantification of ribonucleotides in the genome

**DOI:** 10.1111/gtc.12871

**Published:** 2021-06-02

**Authors:** Tetsushi Iida, Naoko Iida, Jun Sese, Takehiko Kobayashi

**Affiliations:** ^1^ Laboratory of Genome Regeneration Research Center for Biological Visualization The Institute for Quantitative Biosciences (IQB) Tokyo Japan; ^2^ Division of Genome Analysis Platform Development National Cancer Center Research Institute Tokyo Japan; ^3^ Section of Genome Analysis Platform Center for Cancer Genomic and Advanced Therapeutics (C‐CAT) National Cancer Center Research Institute Tokyo Japan; ^4^ Artificial Intelligence Research Center National Institute of Advanced Industrial Science and Technology (AIST) Tokyo Japan; ^5^ Collaborative Research Institute for Innovative Microbiology the University of Tokyo Tokyo Japan; ^6^ Present address: Humanome Lab., Inc. Tokyo Japan

**Keywords:** genome instability, indel, mutation, repair, ribonucleotide, RNase H

## Abstract

Ribonucleotides incorporated in the genome are a source of endogenous DNA damage and also serve as signals for repair. Although recent advances of ribonucleotide detection by sequencing, the balance between incorporation and repair of ribonucleotides has not been elucidated. Here, we describe a competitive sequencing method, Ribonucleotide Scanning Quantification sequencing (RiSQ‐seq), which enables absolute quantification of misincorporated ribonucleotides throughout the genome by background normalization and standard adjustment within a single sample. RiSQ‐seq analysis of cells harboring wild‐type DNA polymerases revealed that ribonucleotides were incorporated nonuniformly in the genome with a 3′‐shifted distribution and preference for GC sequences. Although ribonucleotide profiles in wild‐type and repair‐deficient mutant strains showed a similar pattern, direct comparison of distinct ribonucleotide levels in the strains by RiSQ‐seq enabled evaluation of ribonucleotide excision repair activity at base resolution and revealed the strand bias of repair. The distinct preferences of ribonucleotide incorporation and repair create vulnerable regions associated with indel hotspots, suggesting that repair at sites of ribonucleotide misincorporation serves to maintain genome integrity and that RiSQ‐seq can provide an estimate of indel risk.

## Graphical abstract

Ribonucleotide Scanning Quantification sequencing (RiSQ‐seq) enables absolute quantification of misincorporated ribonucleotides (rNMPs) in the genome by competitive sequencing between rNMPs and backgrounds. The rNMP quantification revealed non‐uniform incorporation and repair activity of rNMP. The Yin‐yang pattern of rNMP repair activity creates vulnerable regions associated with hotspots of insert‐deletion (indel) mutations. RiSQ‐seq can provide an estimate of indel risk.

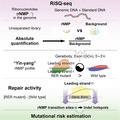

## 1 INTRODUCTION

Endogenous DNA damage, an inevitable consequence of multiple cellular processes, is a relevant source and regulator of genome instability. One type of endogenous damage is misincorporation of ribonucleotides (rNMPs) into the genome during DNA replication or repair (reviewed in Williams et al., 2016). At endogenous concentrations of deoxyribonucleotides and ribonucleotides (dNTPs«NTPs), yeast replicative DNA polymerases frequently misincorporate rNMP in vitro (Nick McElhinny et al., 2010). In wild‐type cells, incorporated rNMPs are efficiently removed by RNase H2‐mediated excision repair (RER)(Nick McElhinny et al., 2010; Sparks et al., 2012). However, hypomorphic mutations in RNase H2 inhibit such repair and are associated with a neuroinflammatory disorder, Aicardi–Goutieres syndrome (Pizzi et al., 2015). Inactivation of RER results in hyper‐accumulation of rNMPs in the *Saccharomyces cerevisiae* and mouse genomes (~10,000 and ~1,000,000 incorporated rNMPs per cell in yeast and mouse, respectively), making rNMPs the most common noncanonical nucleotides in the genome (Nick McElhinny et al., 2010; Reijns et al., 2012). Residual rNMPs cause perturbations in genomic DNA and promote genome instability by mechanisms including ribonucleotide‐triggered abortive DNA ligation, protein–RNA–DNA adducts involving topoisomerases, and replication stress due to polymerase stalling (Clausen et al., 2013; Clausen et al., 2013; Gao et al., 2014; Göksenin et al., 2012; Sparks & Burgers, 2015; Tumbale et al., 2014). By contrast, rNMP incorporation in RER‐proficient cells contributes to efficient removal of mismatches on nascent leading strands by DNA mismatch repair (MMR) (Ghodgaonkar et al., 2013; Lujan et al., 2013). Thus, cells with wild‐type DNA polymerases and RER must maintain an exquisite balance between rNMP incorporation and removal.

Recent efforts to perform rNMP mapping by deep sequencing revealed the nonuniform distribution of rNMPs and the strand‐specific usage of replicative DNA polymerases in the yeast genome (Clausen et al., 2015; Daigaku et al., 2015; Koh et al., 2015; Reijns et al., 2015). All of the previous methods for rNMP mapping reached single‐base resolution mapping by DNA digestion and adaptor ligation at rNMP sites. A previous method, emRiboSeq, digested 5′‐end of rNMPs by RNase H2 treatments (Reijns et al., 2015). In the other methods, HydEn‐seq, Pu‐seq and Ribose‐seq, alkaline digestion allowed to detect 3′‐end position of rNMPs (Clausen et al., 2015; Daigaku et al., 2015; Koh et al., 2015). Furthermore, these methods selectively amplified rNMP detection library by PCR and performed read‐distribution analysis to profile rNMP incorporation in the genome (Clausen et al., 2015; Daigaku et al., 2015; Koh et al., 2015; Reijns et al., 2015). However, PCR amplification of rNMP reads and read‐distribution analysis lost information of background DNA amount without rNMPs. Since estimation of background DNA amounts is essential to quantify actual frequencies of rNMP at each position, methods for precise and comparable quantification of rNMP had not been established.

Although residual rNMPs can contribute to genome instability, the actual frequency of rNMP incorporation and the contribution of RER activity to the maintenance of genome integrity remain unknown. Here, we describe a newly improved sequencing method capable of quantification of rNMPs at base resolution in cells harboring wild‐type DNA polymerases. Comparable quantification of rNMPs provides a simple platform for comparative data analysis, reveals characteristic patterns of rNMP associated with sequence context, quantifies the strand bias of RER activity, and provides a novel metric for rNMP transition, where the rNMP abundance changes rapidly at sites associated with RER inefficiency and indel hotspots. We propose that absolute quantification of endogenous damage represents a new method for evaluation of genome instability.

## 2 RESULTS

### 2.1 RiSQ‐seq: An absolute quantification approach

**FIGURE 1:**
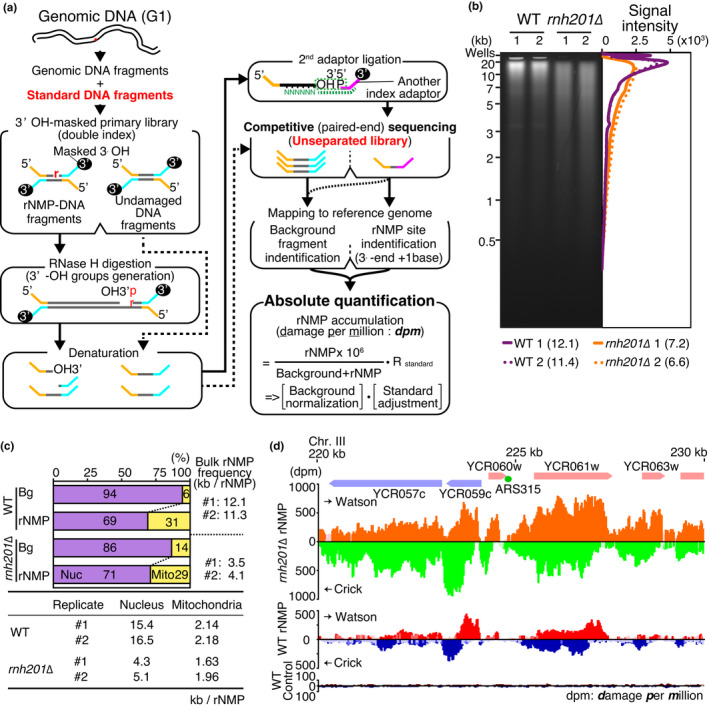
RiSQ‐seq analysis of yeast genomic DNA. (a) Scheme of RiSQ‐seq. Strand‐specific primary libraries are prepared from sheared genomic DNA from G1‐arrested cells and synthetic standard DNAs by ligation with 3′‐OH masked adaptors. After 3′‐OH masking of libraries with ddNTP and terminal‐deoxynucleotidyl transferase, RNase H (HI and HII)‐treated primary libraries are heat‐denatured and ligated with secondary adaptors. Sequencing of unseparated libraries on a MiSeq instrument (Illumina) provides rNMP detection and background reads. Identification of fragments and rNMPs by mapping allows calculation of coverage for rNMP accumulation, adjusting based on a coefficient computed using spiked‐in standards, as damage per million bases (dpm). (b) Representative alkaline gel electrophoresis of genomic DNA from wild‐type (WT) and *rnh201∆* strains (left), with accompanying lane profiles (right). Average DNA lengths are shown in parentheses (kb). (c) Bulk analysis of YPD‐cultured PCR‐free samples by RiSQ‐seq. Sequence composition of background (Bg) and rNMP (rNMP) in PCR‐free libraries is classified according to nuclear (Nuc) and mitochondrial (Mito) origin, with accompanying bulk rNMP frequency (upper panel). Bulk rNMP frequencies in nucleus and mitochondria of replicated samples are shown in the bottom table. Bulk rNMP frequencies of the replicate #1 and #2 are shown. (d) Nonuniform accumulation of rNMP, as determined by RiSQ‐seq analysis. Positions of chromosomes, genes, and the replication origin (ARS315) are shown (top panel). rNMP profiles of *rnh201∆* (orange and green) and WT (orange and green) and control profile of WT sample without RNase H treatment (dark red and navy blue) are shown. rNMP profiles of Watson (upper) and Crick (lower) strands from YPD‐cultured replicate 1 are shown as bar plots with significance of change versus control, as determined by two‐sided Wilcoxon signed‐rank test (faint colored bars: *p* ≥.05). Note that the Crick strand's scale is increasing downward

We developed an absolute quantification sequencing analysis, Ribonucleotide Scanning Quantification sequencing (RiSQ‐seq) by including background and standard detection into the second adaptor ligation method (Figure 1a). To determine the relationship between rNMP incorporation and its repair, it is essential to measure the actual rNMP frequency, allowing estimation of RER activity by simple subtraction of the level of residual rNMPs in the wild type from the level of rNMPs in an RER‐deficient strain (representing the input of rNMP incorporation).

To quantify rNMP, we directly compared sequence reads from an unseparated sequencing library including both background and rNMP‐containing fragments (Figure 1a). RNase H treatment of the primary library after 3′‐hydroxy group masking generated specific targets for secondary adaptor ligation. Secondary adaptor ligation to the 3′‐hydroxy group immediately 5′ of the rNMP allowed detection of the presence of the rNMP (Ding et al., 2015; Reijns et al., 2015). The use of different indexes on primary and secondary adaptors enabled discrimination between background and rNMP detection reads by demultiplexing after paired‐end sequencing. Mapping of paired‐end reads to reference sequences identified insert fragments and rNMP incorporation sites located immediately 3′ adjacent to rNMP detection fragments. The rNMP accumulation level in every analysis bin was computed by normalization of total rNMP against total background coverage counts. To control for bias in sample preparation, the rNMP accumulation level was adjusted by a coefficient, the reciprocal of the detection efficiency of rNMPs, calculated using spiked‐in standards (Figure 1a). By enabling absolute quantification, this approach permits comparison of rNMP frequencies between samples.

First, we tested RiSQ‐seq on a set of synthetic DNA fragments, including both background and rNMP‐containing fragments. To distinguish them, the strands harboring single rNMPs also contained single‐nucleotide polymorphisms (SNPs) at sites proximal to their 3′‐ends. Following sequencing of the primary library, it was possible to determine the rNMP frequency of input DNA fragments by detecting SNPs. Although there was a slight bias among nucleotides, the rNMP detection efficiency (recovery rate) was ~75% (Figure S1a). rNMP detection was completely dependent on RNase H treatment, even at different fragment ratios (Figure S1b). Accordingly, we spiked in these synthetic DNA fragments to serve as standards (Figure 1a). Linear regression models for the spiked‐in standards provided standard curves that could be used to adjust the rNMP accumulation rate (Figure S1c and Table S1).

Genomic DNA of yeast cells arrested in G1 phase, when the genome is ready to enter the next cell cycle after completion of DNA repair, was the ideal sample for comparison of rNMP levels. Alkaline denaturing electrophoresis of genomic DNA prior to alkaline digestion revealed that the genome of *rnh201∆*, an RER‐deficient mutant, was more alkali‐sensitive than that of the wild‐type strain (Figure 1b). This is consistent with previous observations that the *rnh201∆* mutant contains a higher level of rNMPs (Clausen et al., 2015; Lujan et al., 2013; Nick McElhinny et al., 2010; Reijns et al., 2015).

To analyze the intrinsic rNMP level in cells harboring wild‐type DNA polymerases, we quantified rNMPs in the wild type and *rnh201∆* by bulk analysis of RiSQ‐seq libraries. In the wild type, the results yielded rNMP levels similar to those observed in the denaturing gel/alkaline digestion analysis, whereas, in the *rnh201∆* mutant, the rNMP level measured by RiSQ‐seq analysis was slightly higher than the gel analysis (Figure 1b, and the upper panel “Bulk rNMP frequency” of Figure 1c).

The mitochondrial rNMPs accounted for nearly one‐third of total rNMPs in both the wild type and *rnh201∆* (Figure 1c upper panel), and the nucleotide composition of rNMPs in mitochondria showed that purine enrichment in mitochondrial rNMPs was independent of RER status (Figure S3a) (Balachander et al., 2020). The nuclear rNMP frequency was threefold to fourfold higher in *rnh201∆* than in the wild type (Figure 1c bottom). The GC bias of rNMPs in the nuclear genome observed in *rnh201∆* was abolished in the wild type (Figure S3a). These results suggest that the nuclear RER has preference of rNMPs.

**FIGURE 2:**
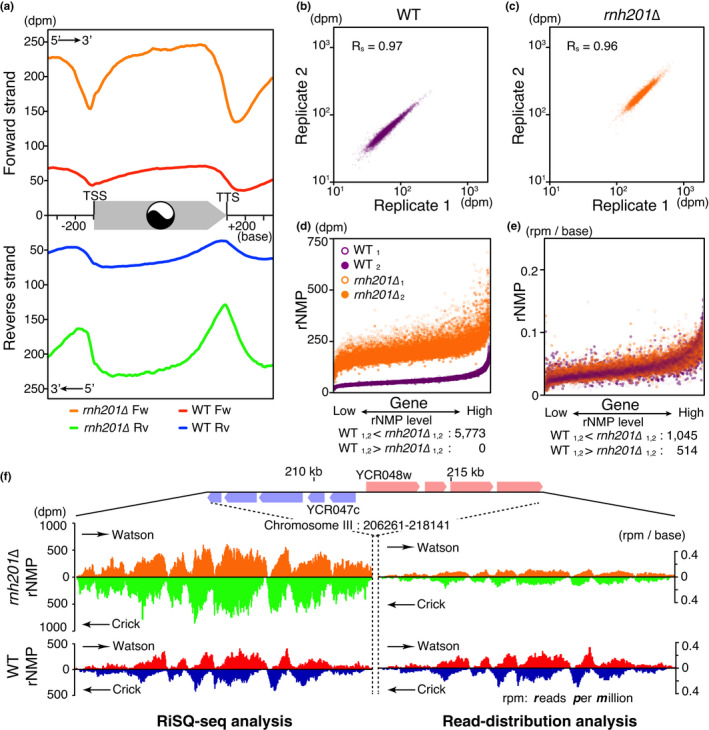
Pattern and range sensitivity of RiSQ‐seq analysis. (a) Metagene profiles of rNMP accumulation on the forward (Fw) and reverse (Rv) strands. rNMPs accumulate in a “yin–yang” pattern at the center of the gene body. Metagene profiles were generated from mean values from YPD‐cultured replicated samples (gene, *N* = 5,828). Note that the reverse strand's scale increases downward. (b and c) Scatter plots to illustrate the reproducibility of RiSQ‐seq. Mean values of rNMP accumulation on the Watson and Crick strands of genes (*N* = 5,828) are plotted for YPD‐cultured replicated samples in the wild type (b) and *rnh201∆* (c). Spearman's rank correlation coefficient rho is shown as Rs in each panel. (d and e) Comparison of rNMP ranges between the wild type and *rnh201∆* by RiSQ‐seq (d) and read‐distribution analysis (e). Genes are sorted from low to high‐rNMP level, as determined by RiSQ‐seq analysis of wild‐type replicates. The number of genes with nonoverlapping rNMP ranges is indicated below (*N* = 5,773). (f) Composition of rNMP accumulation profiles in high‐rNMP cluster region, as determined by RiSQ‐seq and read‐distribution analysis. As in Figure 1d, rNMP profiles of Watson and Crick strands are shown at the same scale for the wild type and *rnh201∆*. For read‐distribution analysis, the rNMP value is shown as reads per million per base (rpm/base)

Because the number of rNMP reads in the PCR‐free library was too low for high‐resolution analysis of the rNMP profile, we amplified rNMP fragments by PCR to compensate. The read coverage of the amplified reads was adjusted to a PCR‐free scale using linear regression models of chromosomal coverage, with a few exceptions, that is, the chromosome XII harboring rDNA repeat and the mitochondrial genome (Figure S2b, Table S1). To avoid PCR amplification bias, we employed PCR‐free library data for mitochondrial rNMP analysis. As shown in Figure 1d and S2a, the profiles exhibited similar, nonuniform, and strand‐specific patterns in the wild type and *rnh201∆*. The strand‐specific broad peaks of rNMP accumulation tended to be separated by intergenic regions, that is, promoters, terminators, and ARSs (Figure 1d and S2a). rNMP accumulation in gene bodies was also observed in Pu‐seq analysis of the *S. pombe* genome (Daigaku et al., 2015), implying that the mechanisms underlying rNMP accumulation are conserved. Strikingly, the metagene profile revealed that rNMPs were preferentially enriched in gene body regions, and that the broad peaks were shifted toward the 3′‐end on each strand; rNMPs accumulated on gene bodies with dyad symmetry in a “yin and yang” pattern (Figure 2a). The nonuniform pattern of rNMP accumulation revealed by absolute quantification in wild‐type G1 cells strongly suggests that, even in wild‐type cells, rNMPs in the genome are carried over into the next cell cycle and represent a potential source of genome instability (reviewed in Caldecott, 2014; Kim et al., 2011; Williams et al., 2016).

### 2.2 RiSQ‐seq enables pattern‐ and range‐sensitive quantification

Recent studies evaluated differences among rNMP profiles using read‐distribution analysis (Clausen et al., 2015; Daigaku et al., 2015; Koh et al., 2015; Reijns et al., 2015). RiSQ‐seq provided reproducible similar profiles of the wild type and *rnh201∆*, and *rnh201∆* had a higher rNMP level than the wild type (Figures 1d, 2a‐d, f). However, relative quantification fails to correctly compare rNMP levels (Figure 2a, e, f): in the RiSQ‐seq analysis, the rNMP range of each gene was always lower in the wild type than in *rnh201∆*, whereas, in the read‐distribution analysis, the rNMP ranges of the two strains overlapped for many genes (Figure 2d, e). These results indicate that RiSQ‐seq analysis has two advantages: it correctly quantifies the rNMP level, and it allows direct comparison of inter‐ or intra‐sample rNMP profiles that would be difficult to achieve by read‐distribution analysis.

### 2.3 GC content is strongly associated with rNMP accumulation

**FIGURE 3:**
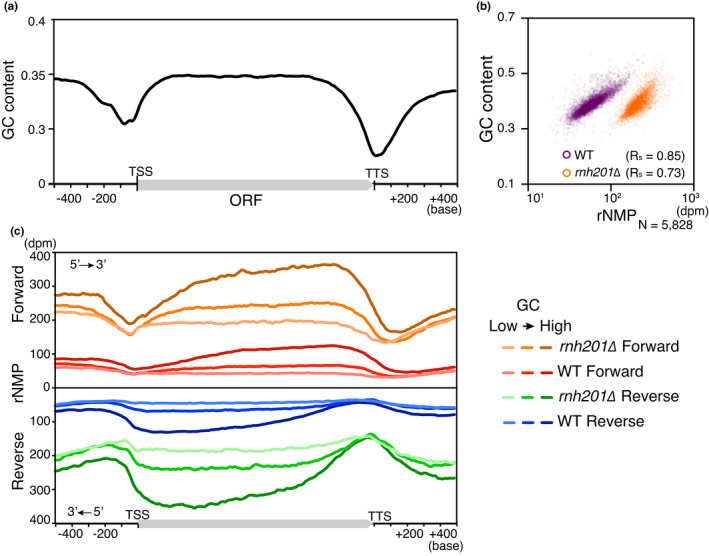
rNMP accumulation associates with GC content. (a) Metagene profile of GC content is shown (gene, *N* = 3,489). (b) Scatter plots for GC content correlation of rNMP. As in Figure 2b and c, rNMP values from YPD‐cultured samples of *rnh201∆* and the wild type are plotted at gene level, with accompanying correlation coefficient R_s_. Nuclear rNMP data are shown. (c) Metagene profiles of rNMP accumulation are shown. The GC level of each gene is classified into quintiles, and metagene profiles of the lowest, middle, and highest fractions (gene, *N* = 1,163 each) are shown

In the previous study by the ribose‐seq, it has been proposed that rNMP accumulation in the mitochondrial genome is preferentially associated with upstream G/C nucleotides and a specific motif (Balachander et al., 2020). In the RiSQ‐seq analysis, the nuclear rNMP accumulates in gene bodies which show higher GC content than their transcriptional promoter and terminator regions (Figures 2a, 3a), implying that the rNMP accumulation associates with high GC content. Consistent with this idea, the rNMP accumulation in gene bodies showed strong correlation with GC content in both wild type and *rnh201∆* (Figure 3b). Furthermore, the yin–yang profile of rNMP level was more pronounced in gene fractions with higher GC content (Figure 3c).

**FIGURE 4:**
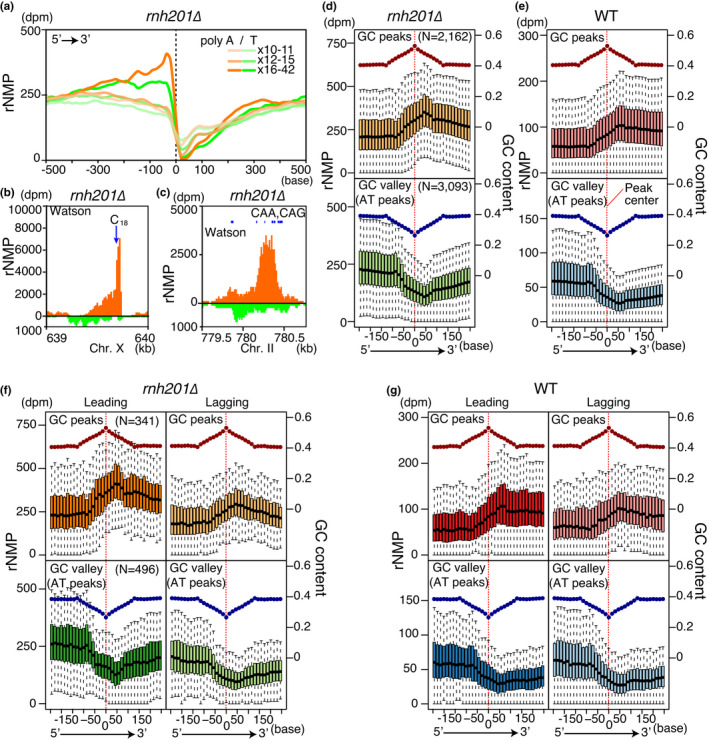
3′‐shifted patterns of local rNMP accumulation. (a) Meta‐analysis around A/T homopolymers (>×10). Profiles of rNMP accumulation in *rnh201∆* around homopolymers (*N* = 2,067) with the indicated length are plotted in the 5′→3′ direction. Dashed line and 0 indicate the centers of homopolymers. (B and C) rNMP profiles around a C stretch (b) and triplet repeats (c) as in Figure 1d. Characteristic repeats in Watson strands are shown. (d–g) Boxplots of rNMP accumulation in the wild type (e and g) and *rnh201∆* (d and f) around GC and AT peaks, with mean GC contents (dots). rNMP distribution around isolated GC (upper panels) and AT peaks (lower panels) (252 bases 5′‐upstream and 3′‐downstream) is shown in 14‐base bins. Red dashed lines show center positions of GC and AT peaks. To show major distribution, upper and lower extremes show one quartile range, and outliers are not shown. In (f) and (g), peaks with high bias of Okazaki fragment coverage rate (OF‐bias >0.9) are selected

Because rNMP levels dropped at TSSs and TTSs (Figure 3a, c), we collected A/T‐stretch sequences and analyzed their rNMP incorporation profiles in *rnh201∆* (Figure 4a). Longer A/T stretches exhibited a more striking transition in the rNMP level in the vicinity of A and T strands. The reduced rNMP level gradually recovered toward the 3′‐end of the transcription unit. By contrast, C_18_ stretches and CAA or CAG trinucleotide repeats had extremely high‐rNMP content (Figure 4b, c). These results confirm that a local sequence affects the rNMP incorporation pattern (Balachander et al., 2020).

To determine whether the rNMP pattern in other sequences responded similarly to repeat sequences, we identified GC and AT peaks throughout the genome. The rNMP distribution around GC and AT peaks formed 3′‐shifted rNMP peaks and valleys, respectively: rNMP peaks and valleys shifted in the 3′ direction by about 50 bases in both the wild type and *rnh201∆* (Figure 4d, e). The 3′‐shifted pattern was observed in both leading and lagging strands (Figure 4f, G). In addition to the shifted pattern, regions 3′ downstream of peaks and valleys showed gradual changes in the rNMP level as inertial patterns (Figure 4d–G). These results suggest that local GC content and inertial nature of rNMP incorporation affect the rNMP accumulation with the yin–yang pattern on gene bodies.

### 2.4 Efficient removal of rNMP on the leading strand

**FIGURE 5:**
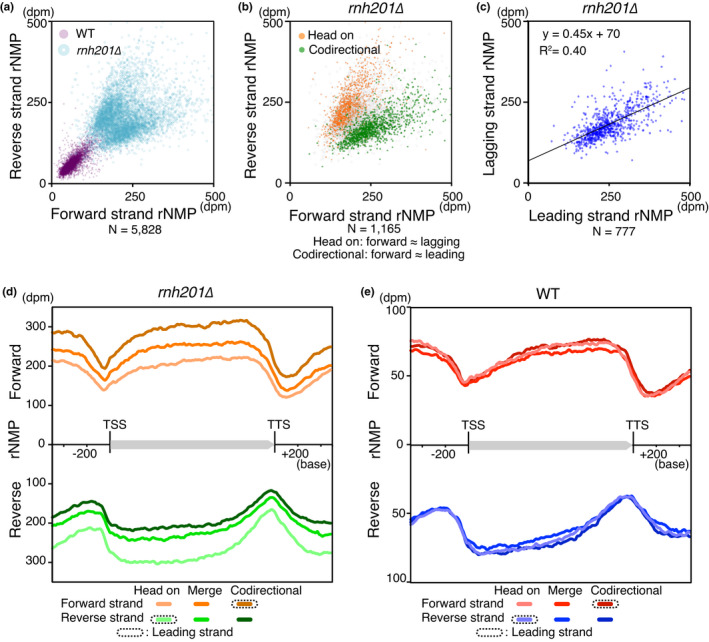
Leading strand preference of rNMP incorporation. (a and b) Scatter plots of gene body rNMP levels in forward and reverse strands of genes. Mean rNMP level of the wild type (a) and *rnh201∆* (a and b) are shown. In (b), genes are classified according to their orientation relative to the replication direction in quintiles of accumulation rate of Okazaki fragments on each strand (OF‐rate), and the highest and lowest fractions are plotted. (c) Leading strand‐biased rNMP incorporation. Mean rNMP levels of genes with high OF‐rate bias (≥0.95) were used for strand analysis (*rnh201∆*). Linear regression model is shown. The difference in rNMP incorporation rate between the leading and lagging strands was estimated from the slope of the linear regression model (1/0.45≈2.2). (d and e) Metagene profiles of rNMP accumulation in *rnh201∆* (d) and the wild type (e). Genes are classified according to OF‐bias for determination of gene orientation. The leading strand profiles are highlighted by dash lines in graph legends

Comparison of gene body rNMP levels between the sense (forward) and template (reverse) strands of transcription in *rnh201∆* revealed a branching pattern that was clearly separated according to gene orientation relative to the direction of replication (Figure 5a, b). Metagene profiles indicated that more rNMP was incorporated in the nascent leading strand than in the lagging strand (Figure 5d). A regression model of gene body rNMP level in *rnh201∆* revealed that the rNMP incorporation rate was 2.2‐fold higher in the leading strand (Figure 5c). Furthermore, the rNMP composition of the leading strand in *rnh201∆* revealed a significantly higher GC preference than that of the lagging strand (Figure S3b). These results were consistent with the in vitro behavior of the major leading strand DNA polymerase ε, which has a strong GC preference and efficiently incorporates rNMPs, in contrast to the major lagging strand DNA polymerase δ (Nick McElhinny et al., 2010).

In contrast to the higher incorporation of rNMP at the leading strand in *rnh201∆* (Figure 5b, d), the residual rNMP levels in the wild type were similar in the leading and lagging strands (Figure 5a, e, S3b). The elimination of strand bias in the wild type implied that RER might have a strong preference for nascent strands.

**FIGURE 6:**
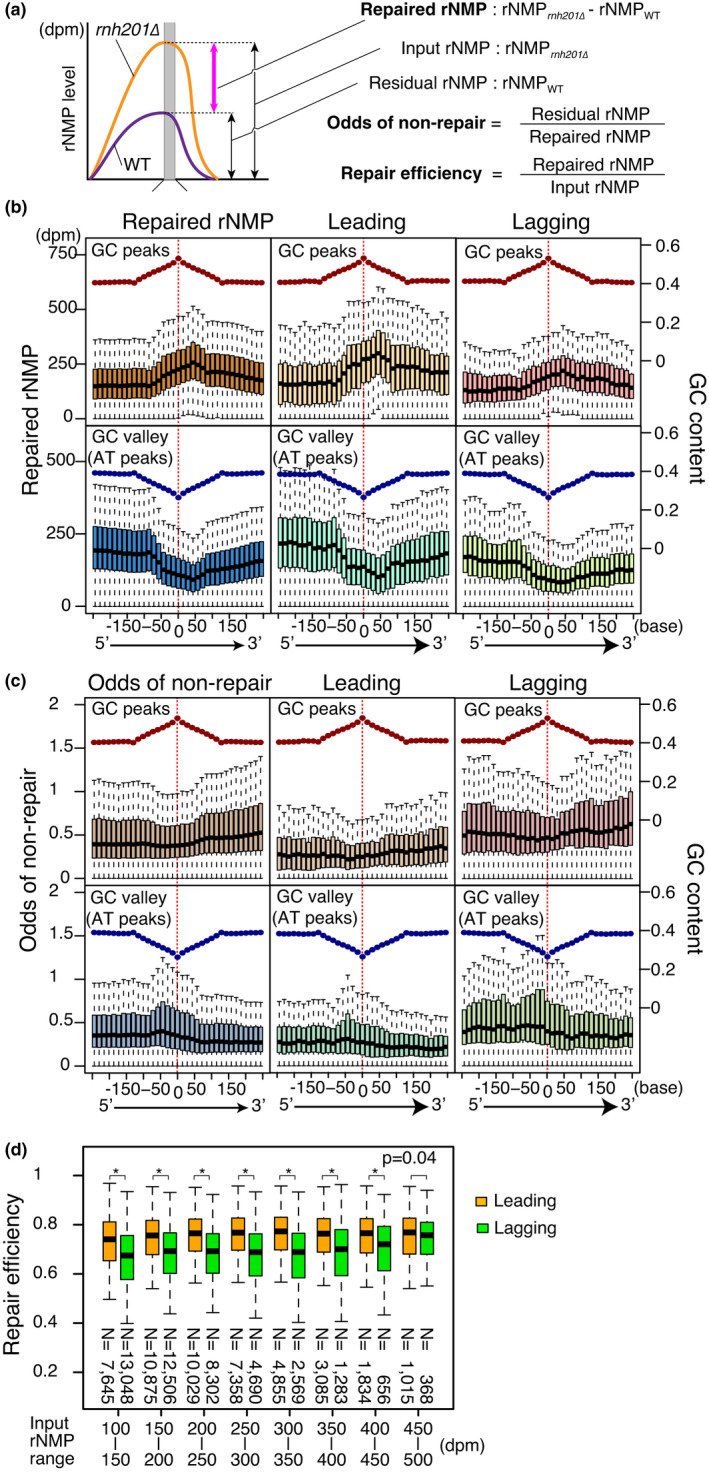
Efficient RER activity on the leading strand. (a) Definition of repaired rNMP, odds of nonrepair, and repair efficiency. (b) Repair efficiencies of leading and lagging strands (*N* = 52,338) were analyzed by linear regression models without intercepts. (b and c) Boxplots of repaired rNMP (b) and odds of nonrepair (c) around GC and AT peaks as in Figure 4d. (d) Repair efficiency of leading and lagging strands at different levels of rNMP input. Repair efficiencies between leading and lagging strands were compared using the two‐sided Mann–Whitney U test. *p < 2.0 × 10^–16^

Because quantification by RiSQ‐seq allowed direct subtraction of the rNMP level, we calculated RER‐repaired rNMP by simple subtraction of residual rNMP from input rNMP. To compare repair efficiency, we also normalized repaired rNMP by input rNMP. Furthermore, we defined the odds of nonrepair, a local index of the difficulty of repair, as the ratio of residual to repaired rNMP (Figure 6a). Although both repair efficiency and odds of nonrepair are essentially equivalent, the latter allows easier comparison of regions in which RER is inefficient. The profiles of repaired rNMPs around GC and AT peaks were related to the input rNMP (Figures 4d, f, 6b). The odds of nonrepair followed a relatively smooth pattern around GC peaks, and 5′‐shifted peaks around AT peaks (Figure 6c). Moreover, the odds were lower on the leading strand than on the lagging strand (Figure 6c). Although input rNMP was higher on the leading strand than on the lagging strand (Figures 4f, 5d), the leading strand exhibited more efficient repair at all tested ranges of input rNMP (rNMP*_rnh201∆_*: 100–500 dpm) (Figure 6d), indicating that RER has a preference for the leading strand independent of input rNMP. The distinct preferences of rNMP incorporation and RER potentially create vulnerable regions in the genome where incorporated rNMP is not efficiently removed.

### 2.5 rNMP transition sites associate with indel mutations

**FIGURE 7:**
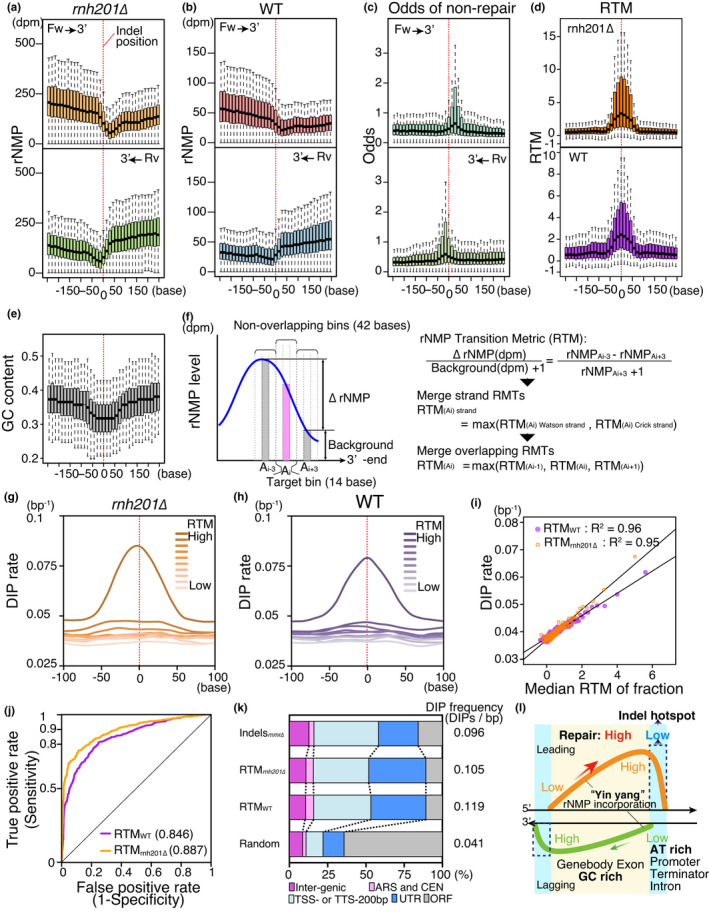
rNMP transition sites are associated with indel mutation. (A and B) rNMP distribution in *rnh201∆* (a) and the wild type (b) around 1,012 indels previously identified in the *msh2∆* cells (Lujan et al., 2014). Red dashed lines indicate indel positions. Forward (upper panel) and reverse (lower panel) strands are classified by OF‐rate at each mutation site, that is, forward indicates the major replication direction. (c) Distribution of odds of nonrepair, as in (a). (d) Distribution of rNMP transition metric (RTM) in *rnh201∆* (upper panel) and the wild type (lower panel). Distribution is shown in the forward strand direction. (E) GC content around indel mutations, as in (d). (f) Definition of rNMP transition metric (RTM). (g and h) Meta profiles of DIP rate around peak fractions. Peaks of *rnh201∆* RTM (g) and wild‐type RTM (h) are fractionated into deciles, and DIP rate around each peak fraction is shown. Distribution on the Watson strand is shown. (i) Relationship of RTM to DIP rate is analyzed by regression analysis. Means of DIP rate in each percentile fraction are plotted. R^2^ of fit is indicated. (j) ROC curve of RTMs for identification of indel mutations. AUCs are also indicated. (k) Annotation composition of indel hotspots predicted by RTMs. Classified annotations of predicted indel hotspots are shown, with accompanying DIP frequency (*rnh201∆*: *N* = 2,339, WT: *N* = 1,519). Previously reported indels in *mmr∆* (*msh2∆*) by Lujan et al. (*N* = 1,012) and randomly selected sites (*N* = 2,339) are also shown as controls. (l) The distinct preferences of incorporation and repair of rNMPs result in indel hotspots. The yin–yang pattern of rNMP incorporation in GC‐rich region spreads over adjacent AT‐rich regions with a preference for the leading strand. The regions of RER inefficiency associate with rNMP transition sites, AT‐rich regions, and indel hotspots

Residual rNMPs could be a source of genomic instability such as spontaneous mutagenesis (reviewed in Caldecott, 2014; Williams et al., 2016). In the RER‐deficient strain, *rnh201∆*, rNMP accumulation throughout the genome is elevated, as is the rate of 2–5‐base deletion mutations in short tandem repeats (Chen et al., 2000; Clark et al., 2011; Kim et al., 2011; Nick McElhinny et al., 2010). To determine whether rNMP accumulation and/or RER inefficiency make important contributions to mutagenesis in cells with wild‐type DNA polymerases, we focused on datasets of spontaneous mutations previously identified in the absence of selective pressure by Lujan et al. (Lujan et al., 2014). rNMP accumulation around the reported indels by Lujan et al. in the mismatch repair (MMR)‐deficient mutant *msh2∆* exhibited a clear transition pattern on both strands near mutation sites, whereas the base‐substitution mutations reported by Lujan et al. and randomly chosen sites in this study exhibited random, flat distribution patterns (Figure 7a, b, S4a–c) (Lujan et al., 2014). Because this transition pattern was also found around AT peaks (Figure 4d, e), indels were associated with low‐GC region (Figure 7e). We also found that indels associated with clear peaks in odds of nonrepair on both strands (Figure 7c). The association among indels, rNMP accumulation, and RER inefficiency suggested that it is not high‐rNMP level, but rather significant RER inefficiency, that increases the risk of indels in cells with wild‐type DNA polymerases. Notably, most of the indels analyzed here were single‐base changes potentially resulting from polymerase slippage, rather than Top1‐mediated 2–5‐base deletions (Kim et al., 2011; Lujan et al., ,^2014^, ^2015^).

Because both rNMP accumulation and odds of nonrepair associate closely with each other, we defined an index for the rNMP transition pattern, the rNMP Transition Metric (RTM), which is calculated using the significance of the reduction in the rNMP level (to analyze RER inefficiency) (Figure 7f). In the RTM profiles of the wild type and *rnh201∆*, the centers of RTM peaks are associated with indels, implying that RTM is a marker of indel risk (Figure 7d). To determine whether RTM is applicable to natural variations among wild‐type yeast strains, we analyzed distributions of deletion–insertion polymorphisms (DIPs) around RTM peaks in 37 *S. cerevisiae* strains (Liti et al., 2009). In both the wild type and *rnh201∆*, the peaks of the highest RTM fractions overlapped extensively with DIP peaks (Figure 7g, h). Furthermore, the median values of fractions of RTM peaks were highly correlated with DIP rate at peak centers (Figure 7i). These results strongly support the idea that RTM is an accurate marker of indel risk in evolutionally diverse.

To evaluate RTM as markers of indel risk, we randomly picked sites without indels or DIPs as negative controls, and combined them with the indels previously identified in *msh2∆* (Lujan et al., 2014). The rate of indels identified (true positive rate: sensitivity) and rate of negative controls identified (false positive rate: 1‐specificity) to all indels and controls at every value of RTM was analyzed by Receiver Operating Characteristic (ROC) curve analysis (Figure 7j). Using this analysis, we evaluated marker accuracy and estimated the thresholds at certain sensitivities. The areas under the ROC curves (AUC) indicated that both RTMs had high sensitivity and specificity (Figure 7j): furthermore, sensitivity over 0.99 of specificity of RTM*_rnh201∆_* and RTM_WT_ was 0.40 and 0.15, respectively. To determine whether RTM peaks identify hotspots of evolutionarily selected DIPs, we analyzed high‐RTM sites. The high‐RTM sites with a specificity of over 0.99 in the ROC curve were associated with DIP‐enriched regions and noncoding regions of the genome, as were indels in *msh2∆* (Figure 7k), suggesting that RTM obtained from RiSQ‐seq analysis is a good marker of indel risk, even in MMR‐proficient cells.

We further analyzed whether the high‐RTM sites are associated with A/T stretches in the genome (>×10, *N* = 2,067) (Figure 4a). About half of the A/T stretches (*N* = 1,081) overlap with the high‐RTM sites of *rnh201∆* (Figure 7k) at a very high DIP rate (0.114). The other A/T stretches that do not overlap with the high‐RTM sites (*N* = 986) showed approximately 1.5‐hold lower DIP rate (0.078) than the high‐RTM overlaps. In contrast, the high‐RTM sites of *rnh201∆* without long A/T stretches (*N* = 1,236) still showed a high DIP rate (0.107). These results suggest that scanning of high‐RTM sites more efficiently predicts indel hotspots than simple search of long A/T stretches in the genome.

## 3 DISCUSSION

In this study, we developed RiSQ‐seq, a system for sequence analysis of rNMP accumulation that achieves absolute quantification of genomic DNA samples by a combination of background normalization and adjustment based on spiked‐in standards. The dataset of high‐resolution profile of absolute rNMP quantification allows analysis of cells harboring wild‐type DNA polymerases and repair systems. In addition, simple subtraction enables direct comparison of profiles between samples, which is difficult to achieve using other methods.

### 3.1 Advantages and applications of absolute quantification

The whole‐genome profile of the absolute quantity of damage provides key information for analyses of repair activity, as well as identification of fragile and/or mutagenic regions of genome. The commonly used technique of read‐distribution analysis obscures fair comparison of the quantity of damage among samples: comparison of similar profile patterns requires knowledge of absolute quantities (Figure 2e–f). Here, we demonstrated that the profiles of rNMP accumulation determined by absolute quantification using RiSQ‐seq can provide RER frequency by simple subtraction, as well as serve as a marker for indel hotspots. The concept of unseparated libraries could also be applied to absolute quantification using other combinations of enzymes and damage, such as formamidopyrimidine DNA glycosylase (FPG) and oxidation‐damaged purines (reviewed in Cooke et al., 2003). We anticipate that analysis of unseparated libraries will be of broad use for studies of many types of endogenous DNA damage that could form a new category of epigenetic marks for the evaluation of genome quality.

### 3.2 The yin–yang pattern of rNMP incorporation

Our RiSQ‐seq analysis revealed a nonuniform distribution of rNMPs, which we refer to as the “yin–yang” pattern. This 3′‐shifted distribution, which is observed at both gene and base scale, has interesting features. (a) The pattern is observed on both leading and lagging strands. (b) rNMP incorporation is closely associated with sequence context. Together, these observations suggest that the mechanism underlying this pattern might involve a common feature of DNA polymerase activity that is affected by sequence context. Locally, the rNMP level is dramatically reduced at A/T stretches, in a length‐dependent manner (Figure 4a). By contrast, C_18_ stretches and CAG trinucleotide repeats are associated with abundant rNMP incorporation (Figure 4b, c). These simple repeats can act as sources of genomic instability by perturbing replication: A stretches, AT dinucleotide, and CAG trinucleotide repeats facilitate pausing of human DNA polymerases in vitro (Le et al., 2015; Walsh et al., 2013). Although the difference between the mechanisms of replication pausing at AT‐rich repeats and G/C‐containing repeats remains unclear, sequence context might directly regulate DNA polymerase fidelity via local replication status, for example, speed of the replication fork and/or 5′→3′ DNA synthesis.

### 3.3 rNMP regulation is associated with genome instability in cells with wild‐type DNA polymerases

Although residual rNMPs represent an endogenous threat of genome instability (reviewed in Williams et al., 2016), our rNMP quantification reveals that significant amounts of rNMPs remain in the G1‐phase genome even in RER‐proficient cells with wild‐type DNA polymerases. The nonuniform pattern of residual rNMPs indicates that specific regions of the genome are at elevated risk of genomic instability on the template strand, even in wild‐type cells. High‐rNMP genes are strong candidates for natural hotspots of genomic instability.

Although it is still possible that rNMP transition and indel hotspots are independently coincidental in response to the similar DNA context, our findings demonstrate that RER efficiency and rNMP transition sites, but not the level of rNMP accumulation, are well correlated with indel mutations. The inertial delay and GC preference of rNMP incorporation suggest that attenuation of DNA polymerase fidelity gradually increases on gene bodies or exons, reaching its peak in AT‐rich regions (Figure 7l). Furthermore, inefficient RER also promotes mutational risk in such regions adjacent to AT‐rich domains (Figures 6c, 7c, e, L). Most of the single‐base indels might result from polymerase slippage rather than Top1‐dependent error‐prone repair (Kim et al., 2011; Lujan et al., 2015). Such indels, which are often associated with long homopolymer sequences, are efficiently suppressed by MMR, and RER also contributes to suppression of single‐base indels (Lujan et al., 2013). Because MMR and RER cooperatively suppress mismatches and slippages that escape proofreading (Ghodgaonkar et al., 2013; Lujan et al., 2013), regions exhibiting RER inefficiency with high‐RTM values are vulnerable to error accumulation, and can thus become indel hotspots. Furthermore, a previous study showed by mutation bias analysis that replication fidelity is higher on the leading strand than the lagging strand (Lujan et al., 2014). In this study we also found that RER is more efficient on the leading strand at sequences with a wide range of rNMP incorporation. This suggests that RER and rNMP incorporation, rather than being source of mutagenic damage, supply periodic postreplicative repair during the cell cycle.

Estimation of RER efficiency by RiSQ‐seq analysis requires both rNMP quantification and comparison between RER‐proficient and ‐deficient cells by RiSQ‐seq analysis. However, either alone is sufficient to compute RTM, which is closely associated with RER inefficiency in yeast. Because RER is conserved among species (Nick McElhinny et al., 2010; Reijns et al., 2012), the simplified approach using the RTM parameter could be applied to whole‐genome surveys of indel hotspots in other organisms. Moreover, because RiSQ‐seq analysis is a self‐sufficient technique requiring simple starting materials (i.e., purified genomic and standard DNAs), it can provide genomic parameters related to genome instability in various cell types, such as cancer cells or stem cells, for evaluation of genome quality.

## 4 EXPERIMENTAL PROCEDURES

### 4.1 Yeast strains and cell culture

The wild‐type strain BY4741 (YTT0003: *MAT*a *bar1∆::hphMX trp1::p404–BrdU–Inc*(*TRP1*) *his3∆1 ura3∆0 leu2∆0 met15∆0*) and the RER‐deficient mutant strain *rnh201∆* (YTT0004: YTT0003 with *rnh201∆::kanMX*) were exponentially grown in 1 L of YPD [1% Bacto Yeast Extract (BD), 2% Bacto Peptone, and 2% Glucose (Wako)] or low‐glucose media containing 0.05% Glucose to a density of ~1 × 10^7^ cells/ml, and then arrested at G1 by addition of alpha‐factor at a final concentration of 5 µg/ml, followed by incubation for 2.5 hr at 30°C with shaking (222 rpm) in an INNOVA43 shaker. Cultures were stopped by addition of 0.1% sodium azide (final concentration). The detailed protocols for genomic DNA preparation and Alkali‐denaturing agarose gel analysis are provided in Supplemental Methods.

### 4.2 RiSQ‐seq library preparation and sequencing

Genomic DNAs and standard DNAs, sheared to 400–450 bp, were used for primary library preparation with TruSeq HT compatible adaptors (Table S2). After 3′‐end masking, primary libraries were treated by RNase HI and HII (NEB) at 37°C for 18 hr. Heat‐denatured RNase H–treated libraries were ligated with secondary adaptors at 20°C for 18 hr using the KAPA library preparation kit. After successive purification with AMPure XP beads, complementary strands were synthesized with Bst DNA polymerase (NEB) and purified. Aliquots of PCR‐free libraries (5 ng) were PCR‐amplified with TruSeq HT i5 and TruSeq HT 701 specific primers for 10–22 cycles. Purified libraries (PCR‐free, 27 pM; PCR‐amplified, 15 pM) were loaded onto an Illumina MiSeq (75 bp ×2 paired‐end sequencing) to obtain over 10 million paired reads of data. The detailed protocol for RiSQ‐seq library preparation is provided in Supplemental Methods (Appendix S1).

### 4.3 Data processing

All analyses were performed on rNMP accumulation frequency data. Using these values, we estimated repair frequency by subtracting rNMP levels in the wild type from those in *rnh201∆*. Details regarding data processing are provided in Supplemental Methods (Appendix S1).

### 4.4 Data access

The sequencing data are deposited at Gene Expression Omnibus (GEO) under accession number GSE85130.

## AUTHOR CONTRIBUTIONS

T.I. designed this study. T.I. conducted all the experiments. T.I. performed data analysis with support from N.I. and J.S. T.I. and T.K. wrote the manuscript with contribution from all the authors.

## Supporting information

Supplementary Material
