# Supplementary material for: Evaluation of repair activity by quantification of ribonucleotides in the genome
Source: Genes Cells. 2021 Jun 2;26(8):555–69. doi: 10.1111/gtc.12871 (PMC8453711; doi:10.1111/gtc.12871)
Supplement: Supplementary file 1 — Supplementary Material [file GTC-26-555-s001.pdf]

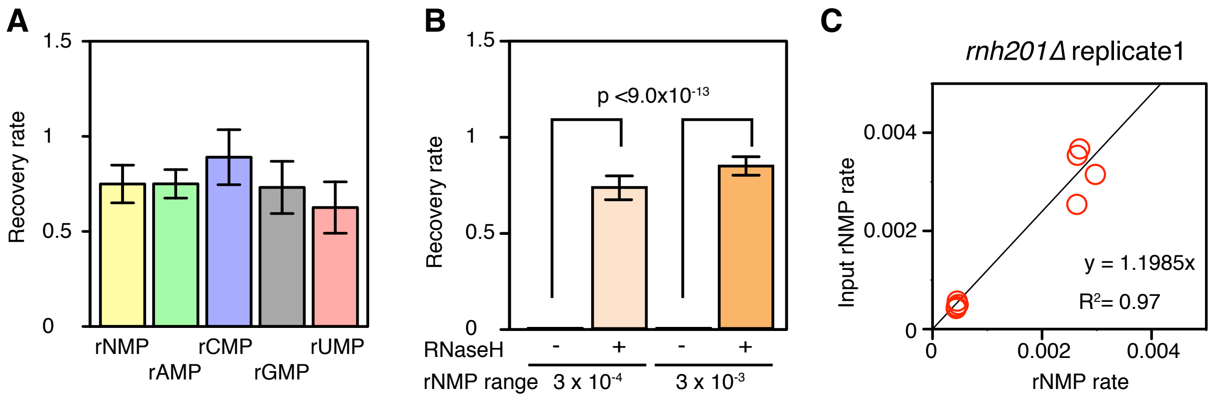

Supplemental Figure S1. Standard fragment analysis by RiSQ-seq
(A) rNMP-detection efficiency (recovery rate) by RiSQ-seq. Each ribonucleotide in synthetic DNA fragments (N=8) was detected by RiSQ-seq. Error bars indicate standard error of the mean (SEM).
(B) rNMP-detection efficiency at different concentrations of rNMP standards. RNase H treatment status and standard concentrations are indicated below. Mock treatment (N=6) and RNase H–treated samples (N=8) were compared by two-sided Mann–Whitney U test. (C) Example of standard curve generated from spiked-in standards (*rnh201Δ* replicate 1). Linear regression model without intercept is shown.

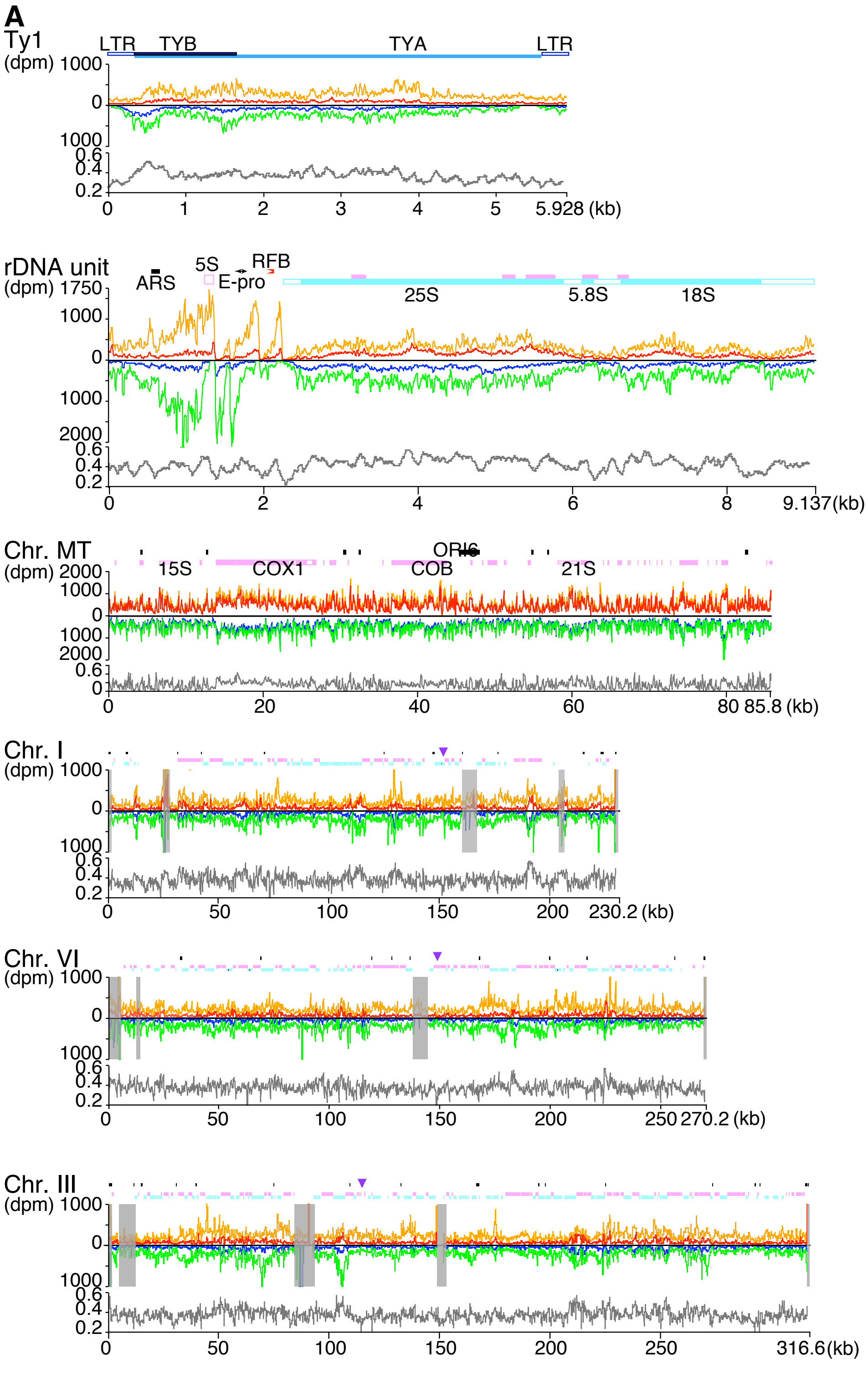

Supplemental Figure S2. (i) rNMP profiles of the yeast genome.

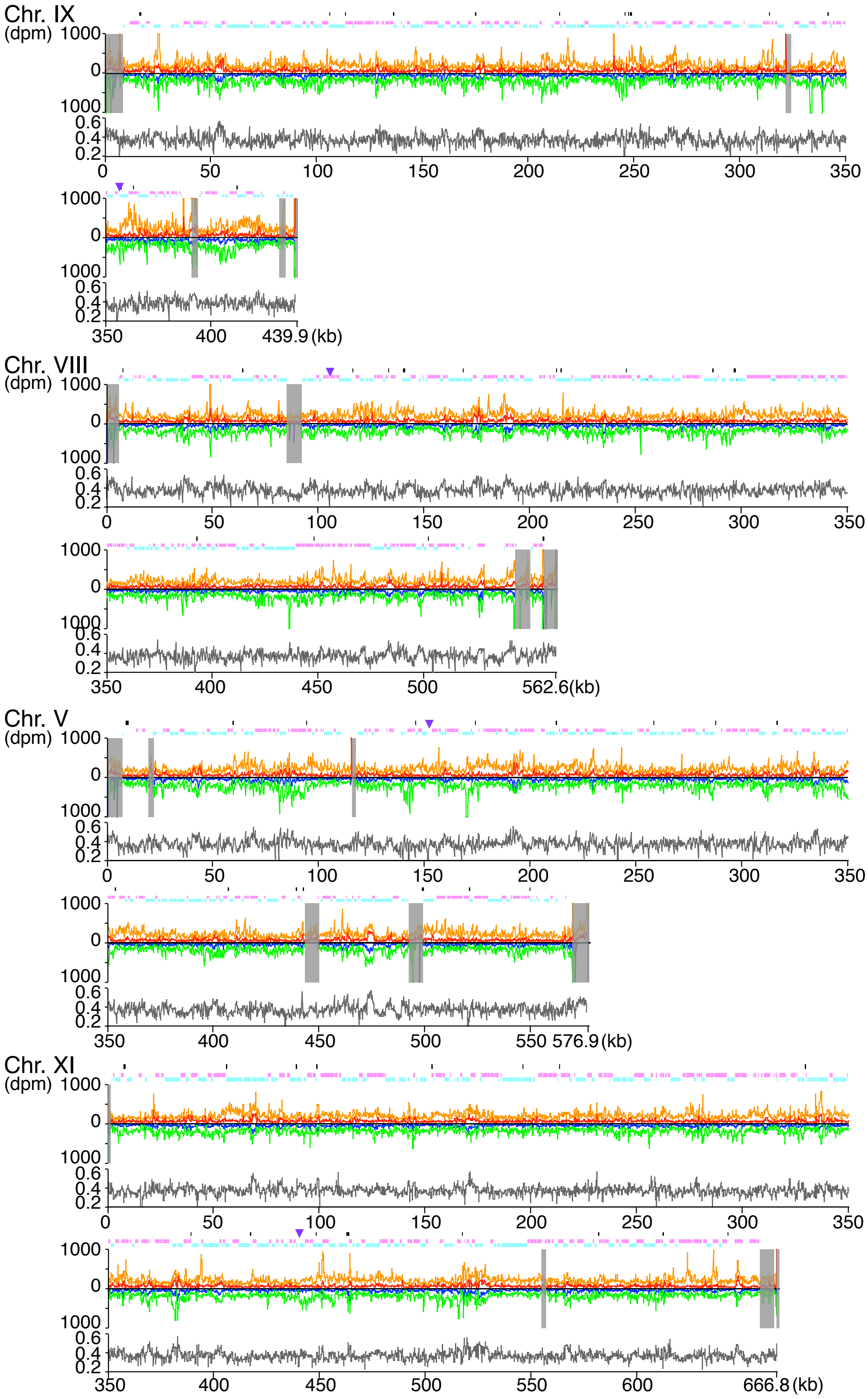

Supplemental Figure S2. (ii) rNMP profiles of the yeast genome.

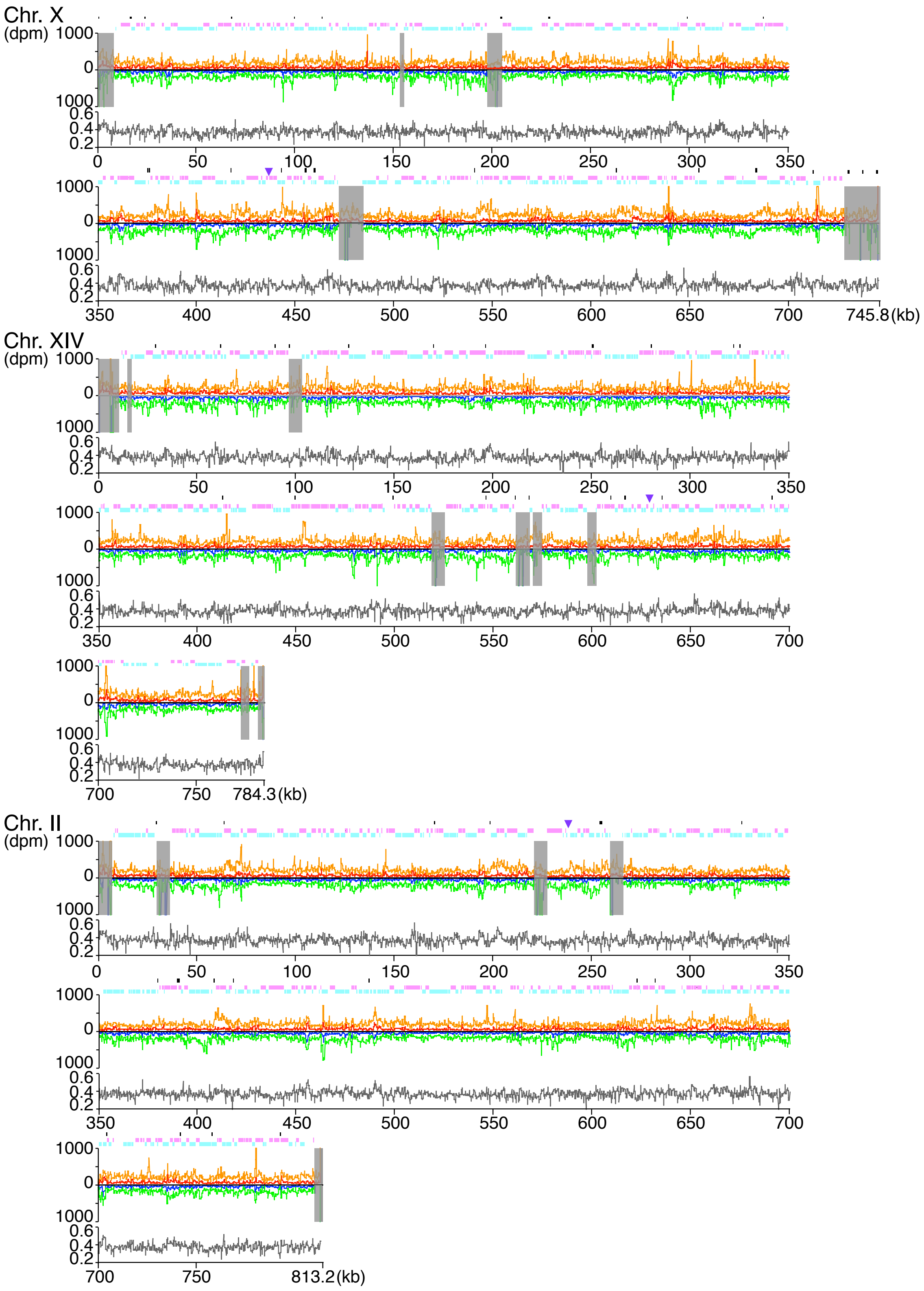

Supplemental Figure S2. (iii) rNMP profiles of the yeast genome.

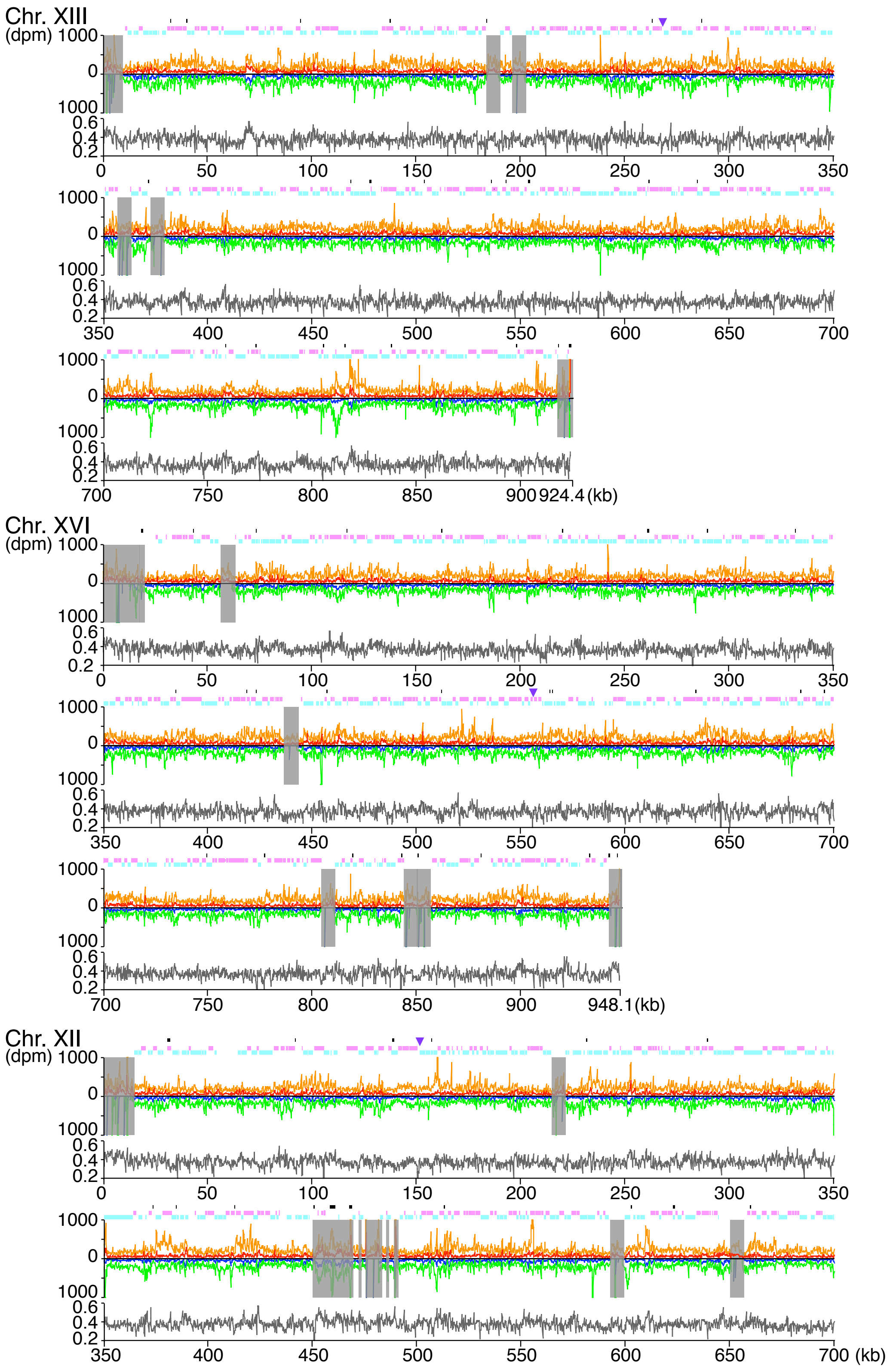

Supplemental Figure S2. (iv) rNMP profiles of the yeast genome.

(Continue)

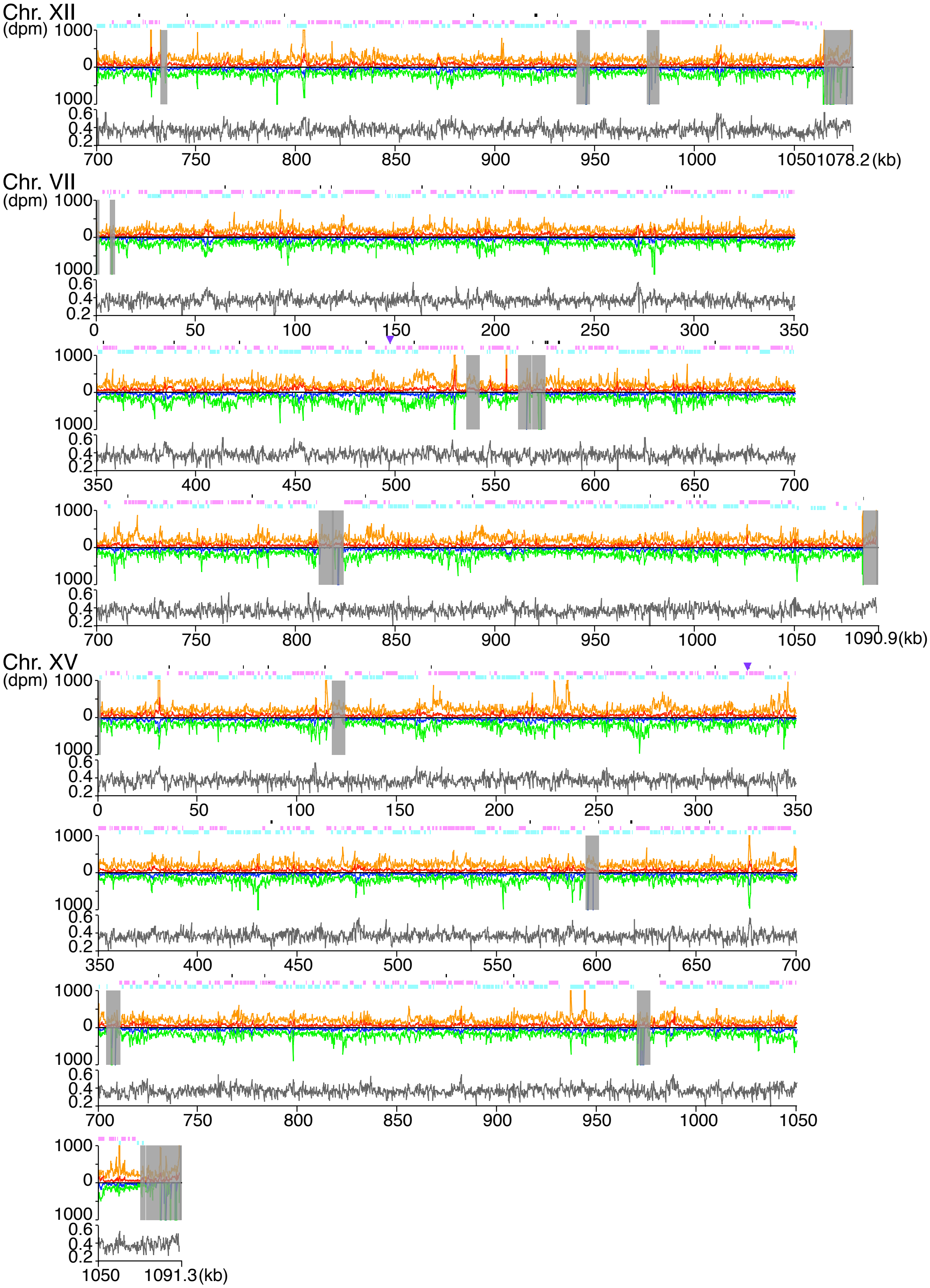

Supplemental Figure S2. (v) rNMP profiles of the yeast genome.

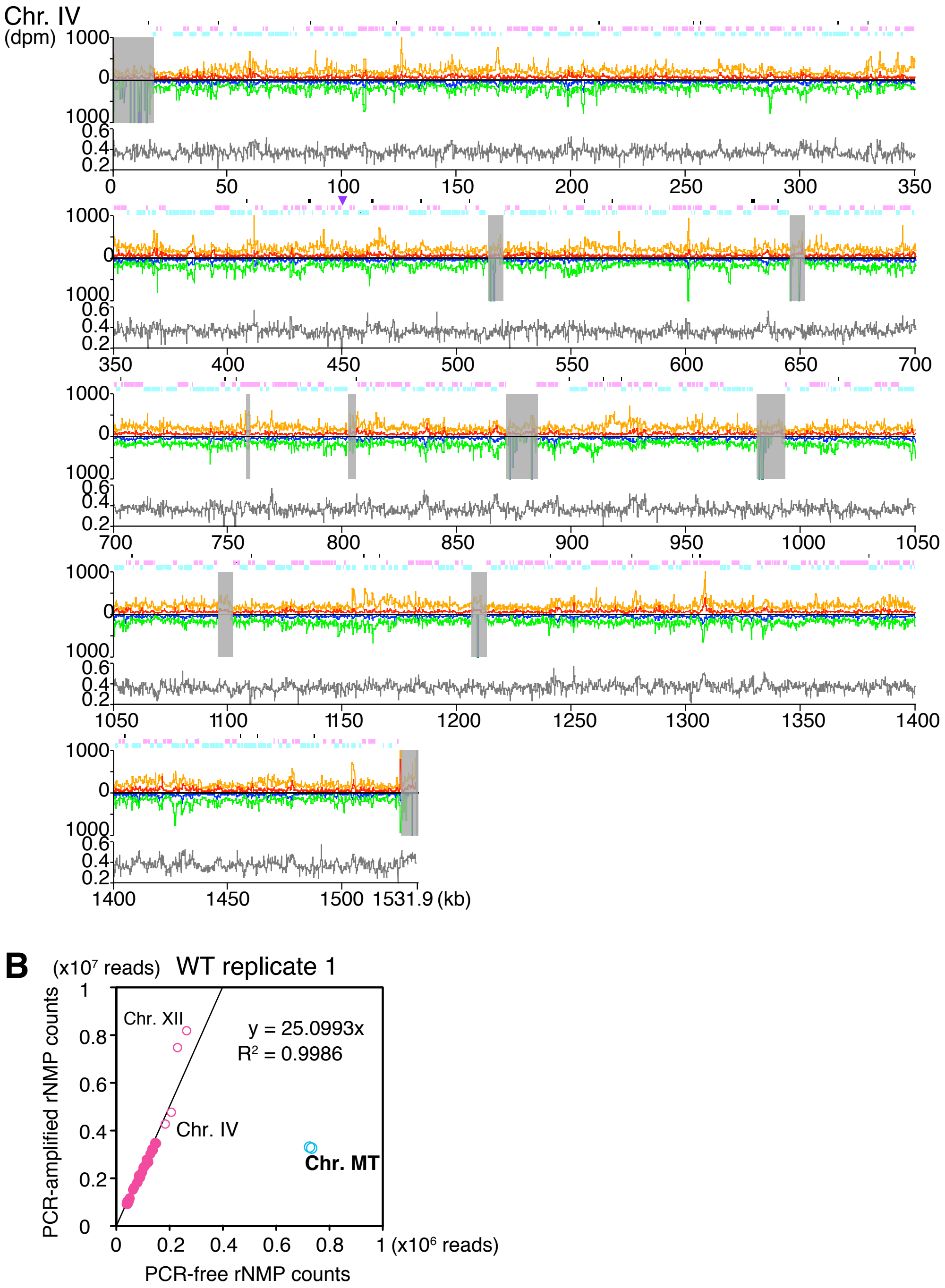

Supplemental Figure S2. (vi) rNMP profiles of the yeast genome.
(A) rNMP quantification profiles of each chromosome and element. As in Figure 1*G*, each top panel shows annotations: boxes indicate genes on the Watson strand (magenta) or Crick strand (cyan) and replication origins (black), and triangles indicate centromeres. Each middle panel shows a line plot of rNMP profiles: Watson strand profiles are depicted as red (wild type) and orange (*rnh201Δ*) lines, and Crick strand profiles are depicted as blue (wild type) and green (*rnh201Δ*) lines. For chromosomes, 42-base bin data are plotted, and, for rDNA repeat unit, 21-base bin data are plotted. In the Ty1 rNMP profiles, rNMP values in 21-base bins are calculated from aligned Ty1 elements by Clustal W. Each bottom panel indicates GC-content profile and chromosomal position. All data were obtained from cells cultured in YPD. Regions with mapping bias that were not included in the quantification analysis are covered with gray boxes. (B) Scatter plot of read counts in chromosomal strands: PCR-amplified rNMP reads vs PCR-free rNMP reads (wild-type replicate 1 of YPD-cultured cells). Linear regression model without intercept is shown. Open circles indicate data not used for regression analysis.

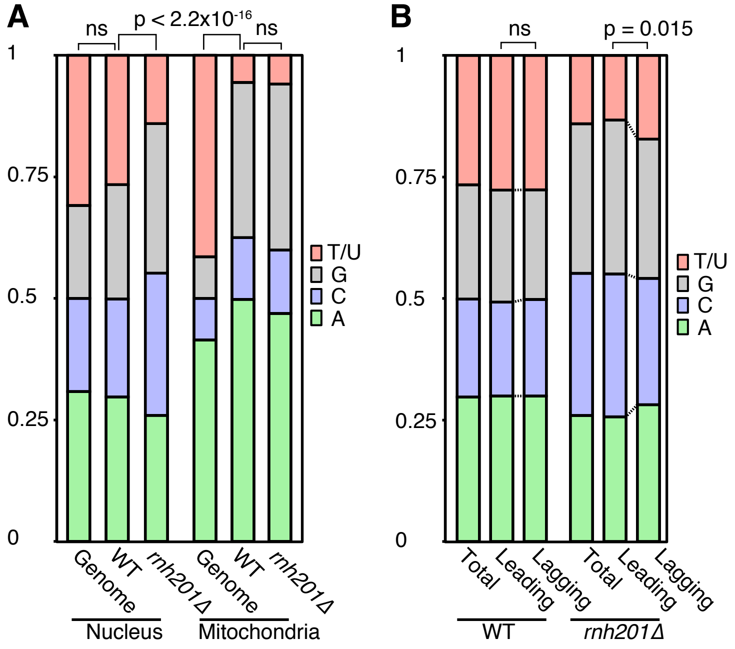

Supplemental Figure S3. Nucleotide composition of genome and rNMPs.
(A) Nuclear and mitochondrial nucleotide composition is shown. (B) Difference between leading and lagging strands is shown. Significance of differences was evaluated by two-sided Chi-squared test (N=500). "ns" indicates not significant ($p \geq 0.05$). Average composition was analyzed in PCR-free data obtained from YPD-cultured cells.

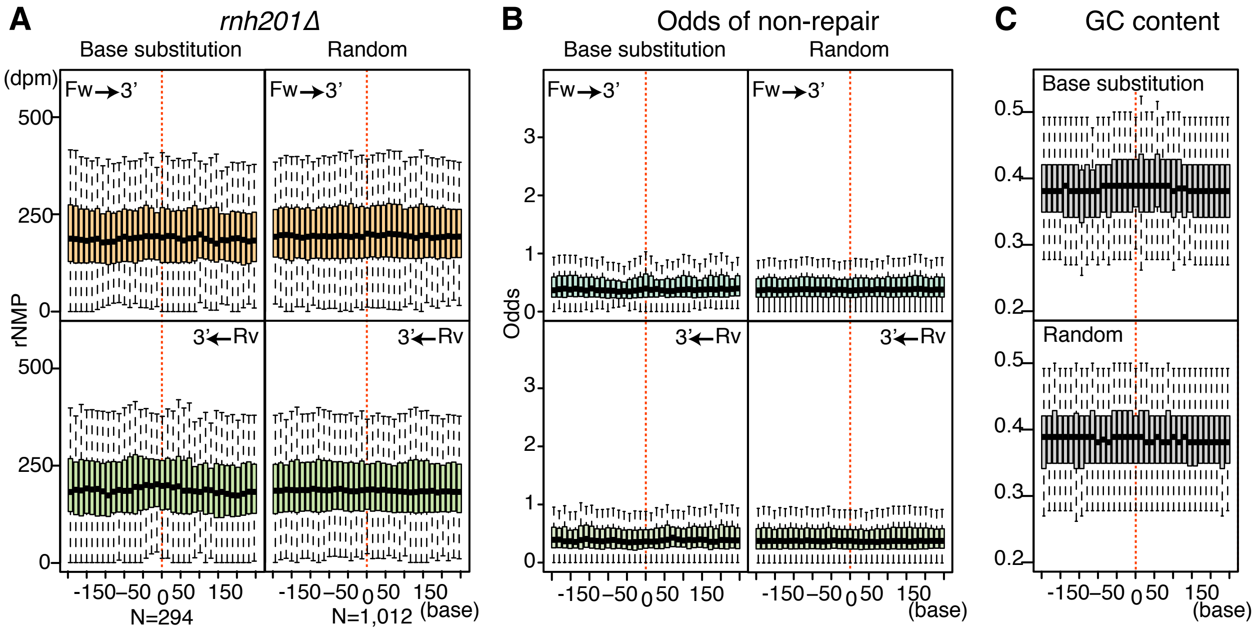

Supplemental Figure. S4. Relationship between rNMP and mutations.
(A and B) Distribution of rNMP incorporation and odds of non-repair around base-substitution mutations previously identified by Lujan et al. (Lujan et al., 2014) and random positions selected in this study. rNMP values and odds of non-repair are plotted as in Figure 7*A* and *C*. Red dashed lines indicate positions of base-substitution mutations and randomly selected sites, respectively. (C) Distribution of GC content around base-substitution mutations (upper panel) and random positions (lower panel) are shown as in Figure 7*E*.

Supplemental Table S1. RiSQ-seq summary

Genotype	Sample#	Culture condition	RNaseH treatment	PCR amplification	Index type	Total read pair counts*	Uniquely mapped pair **	Standard fragment reads**	Correction factor for PCR amplification†	Correction factor of efficiency‡	GEO sample accession
				-	Background	13,630,659	11,254,265	1,378,695	§86.03		GSM2258087
WT	1	YPD_2%	-	-	Nick	332,886	130,042	7,989	NA	1.12614	GSM2258088
				+	Nick	11,307,271	7,807,239	ND	58.2		GSM2258089
				-	Background	15,684,451	12,545,102	1,705,675	NA		GSM2258090
WT	1	YPD_2%	+	-	rNMP	1,004,580	461,014	27,774	NA	1.232	GSM2258091
				+	rNMP	12,247,345	9,001,014	ND	25.1		GSM2258092
				-	Background	17,567,790	14,600,321	1,798,038	§90.54		GSM2258139
WT	2	YPD_2%	-	-	Nick	439,511	160,757	10,798	NA	1.11953	GSM2258140
				+	Nick	22,475,038	12,423,442	ND	76.34		GSM2258141
				-	Background	15,976,315	12,785,442	1,777,450	NA		GSM2258093
WT	2	YPD_2%	+	-	rNMP	981,284	449,025	28,408	NA	1.227	GSM2258094
				+	rNMP	14,083,568	10,476,477	ND	30.11		GSM2258095
				-	Background	17,553,606	14,547,816	1,734,770	§99.68		GSM2258096
WT	1	YPD_0.05%	-	-	Nick	325,774	144,863	8,571	NA	1.16073	GSM2258097
				+	Nick	3,861,113	2,934,890	ND	19.66		GSM2258098
				-	Background	18,757,284	15,456,797	2,066,850	NA		GSM2258099
WT	1	YPD_0.05%	+	-	rNMP	966,929	468,319	30,794	NA	1.271	GSM2258100
				+	rNMP	12,314,446	9,163,778	ND	27.41		GSM2258101
				-	Background	18,222,996	15,767,703	1,332,763	NA		GSM2258102
WT	2	YPD_0.05%	+	-	rNMP	926,059	518,543	25,532	NA	1.094	GSM2258103
				+	rNMP	9,541,917	7,643,518	ND	17.73		GSM2258104
				-	Background	23,225,675	18,770,835	2,426,490	§79.27		GSM2258105
rnh201Δ	1	YPD_2%	-	-	Nick	331,462	232,405	11,207	NA	1.24296	GSM2258106
				+	Nick	10,833,399	8,203,143	ND	35.57		GSM2258107
				-	Background	25,553,810	20,725,612	2,449,827	NA		GSM2258108
rnh201Δ	1	YPD_2%	+	-	rNMP	2,974,115	2,414,735	38,640	NA	1.325	GSM2258109
				+	rNMP	21,917,374	19,194,751	ND	10.15		GSM2258110
				-	Background	23,677,340	19,394,608	2,505,579	§61.04		GSM2258142
rnh201Δ	2	YPD_2%	-	-	Nick	460,710	314,054	26,127	NA	1.12266	GSM2258143
				+	Nick	25,087,487	17,856,282	ND	62.59		GSM2258144
				-	Background	18,481,879	15,012,486	1,871,458	NA		GSM2258111
rnh201Δ	2	YPD_2%	+	-	rNMP	2,090,303	1,656,126	38,215	NA	1.199	GSM2258112
				+	rNMP	19,790,457	14,299,851	ND	11.16		GSM2258113
				-	Background	21,911,399	18,485,746	2,138,773	§87.85		GSM2258114
rnh201Δ	1	YPD_0.05%	-	-	Nick	314,796	205,247	7,928	NA	1.12394	GSM2258115
				+	Nick	11,542,120	8,405,130	ND	40.48		GSM2258116
				-	Background	21,296,923	17,293,117	1,921,117	NA		GSM2258117
rnh201Δ	1	YPD_0.05%	+	-	rNMP	2,629,093	1,898,301	34,717	NA	1.197	GSM2258118
				+	rNMP	15,850,172	13,923,703	ND	9.032		GSM2258119
				-	Background	20,416,036	16,752,918	1,824,097	NA		GSM2258120
rnh201Δ	2	YPD_0.05%	+	-	rNMP	2,080,726	1,678,343	32,937	NA	1.224	GSM2258121
				+	rNMP	17,696,569	15,722,388	ND	10.91		GSM2258122
phiX174RF1	1	NA	-	-	Input	1,310,642	519,409	NA	NA	NA	GSM2258123
phiX174RF1	2	NA	-	-	Input	1,459,848	513,763	NA	NA	NA	GSM2258124
phiX174RF1	3	NA	-	-	Input	1,557,333	644,851	NA	NA	NA	GSM2258125
phiX174RF1	4	NA	-	-	Input	1,385,753	590,966	NA	NA	NA	GSM2258126
phiX174RF1	1	NA	+	-	Background	3,410,116	1,026,462	NA	NA	NA	GSM2258127
				-	rNMP	94,158	12,657	NA	NA	NA	GSM2258128
phiX174RF1	2	NA	+	-	Background	4,000,598	934,669	NA	NA	NA	GSM2258129
				-	rNMP	81,800	12,661	NA	NA	NA	GSM2258130
phiX174RF1	3	NA	+	-	Background	3,972,961	1,153,978	NA	NA	NA	GSM2258131
				-	rNMP	105,037	16,289	NA	NA	NA	GSM2258132
phiX174RF1	4	NA	+	-	Background	3,530,225	1,137,625	NA	NA	NA	GSM2258133
				-	rNMP	71,366	14,563	NA	NA	NA	GSM2258134
phiX174RF1	1	NA	-	-	Input	2,922,981	1,679,072	NA	NA	NA	GSM2258135
phiX174RF1	2	NA	-	-	Input	2,445,490	1,073,203	NA	NA	NA	GSM2258136
phiX174RF1	3	NA	-	-	Input	1,879,045	1,024,182	NA	NA	NA	GSM2258137
phiX174RF1	4	NA	-	-	Input	1,762,013	1,016,091	NA	NA	NA	GSM2258138

* For STD samples, the number of Read2 (reverse read) is shown.

** For STD samples, the number of mapped Read2 (reverse read) is shown.

† Correction factor of PCR reads for PCR-free scale adjustment.

§ Values at background read sample indicate correction factor of background 3'-shifted end for control analysis.

‡ $R_{standard}$ in Figure 1A. Correction factors from rNMP detection efficiency of spiked-in standerds for RNase H treated sample.

Supplemental Table S2. Primer list

Primer	Sequence	Purpose
ChrIII206kb_DraIFw	5'-AAACTTATTAACCAGATAGTAAACAAAATCATGAAAC-3'	Fragment, Probe
ChrIII214kb_DraIRv	5'-AAAAACGCGGTGAAATTTTACCTTGTAAGAACGTC-3'	Fragment
ChrIII207kb_ProbeRv	5'-TGTCCTAACAGGGTTTCATGCAATATTTGCAAGAATG-3'	Probe
ChrXII848kb_BglIIFw	5'-GATCTATCACCCATGATTTGGTCACCAGTAAAAAC-3'	Fragment, Probe
ChrXII856kb_BglIIRv	5'-TTACCACACCTCGTCCACATACTTGTTGTTAATG-3'	Fragment
ChrXII849kb_ProbeRv	5'-GATCCGGTTACCGAGGCTGTATCAAAGGTACC-3'	Probe
STDf1Fw	5'-ATAACGTTTATGTTGGTTTCATGGTTTCCTAGGGAGTTTT ATCGCTTCCATGACGCAGAAG-3'	STD fragment PCR
STDf1Rv	5'-GAACACTCATCCTTAATACCTTTCTTTCCAAGGACTTGAC TCATGATTTCTTACCTATTAG-3'	STD fragment PCR
STDf2Fw	5'-ATAACGTTTATGTTGGTTTCATGGTTTCCTAGGGTACTTAT TCGCCACCATGATTATGACC-3'	STD fragment PCR
STDf2Rv	5'-GAACACTCATCCTTAATACCTTTCTTTCCAAGGCCAGCAT TAACCGTCAAACTATCAAAAT-3'	STD fragment PCR
STDrA	5'-CTAGAGAACCATGAAACCAACrATAAACGTTAT-3'	STD-rNMP adaptor
STDrA_Rv	5'-ATAACGTTTATGTTGGTTTCATGGTTCT-3'	STD-rNMP adaptor
STDrG	5'-CTTGTCAAGAAAGGTATTAAGrGATGAGTGTTC-3'	STD-rNMP adaptor
STDrG_Rv	5'-GAACACTCATCCTTAATACCTTTCTTGA-3'	STD-rNMP adaptor
STDrC	5'-CTAGACAACCATGAAACCAArCATAAACGTTAT-3'	STD-rNMP adaptor
STDrC_Rv	5'-ATAACGTTTATGTTGGTTTCATGGTTGT-3'	STD-rNMP adaptor
STDrU	5'-CTTGTGAAGAAAGGTATTAAGGArUGAGTGTTC-3'	STD-rNMP adaptor
STDrU_Rv	5'-GAACACTCATCCTTAATACCTTTCTTCA-3'	STD-rNMP adaptor
TruHTAdaptor501IddT	5'-AmMC6-TAATGATACGGCGACCACCGAGATCTACACTATA GCCTACACTCTTTCCCTACACGACGCTCTTCCGATC*T-3'	TruHT adaptor
TruHTAdaptor502IddT	5'-AmMC6-TAATGATACGGCGACCACCGAGATCTACACATAG AGGCACACTCTTTCCCTACACGACGCTCTTCCGATC*T-3'	TruHT adaptor
TruHTAdaptor503IddT	5'-AmMC6-TAATGATACGGCGACCACCGAGATCTACACCCTA TCCTACACTCTTTCCCTACACGACGCTCTTCCGATC*T-3'	TruHT adaptor
TruHTAdaptor504IddT	5'-AmMC6-TAATGATACGGCGACCACCGAGATCTACACGGCT CTGAACACTCTTTCCCTACACGACGCTCTTCCGATC*T-3'	TruHT adaptor
TruHTAdaptor702TddC	5'-Phos-GATCGGAAGAGCACACGTCTGAACTCCAGTCACTC CGGAGAATCTCGTATGCCGTCTTCTGCTT*G-3AmMO-3'	TruHT adaptor
TruHTAdaptor701_ss	5'-Phos-AGATCGGAAGAGCACACGTCTGAACTCCAGTCACA TTACTCGATCTCGTATGCCGTCTTCTGCTT* G-3AmMO-3'	Secondary adaptor
ssLigAdaptor-i7short	5'-ACGTGTGCTCTTCCGATCTNNNNNN-3AmMO-3'	Secondary adaptor
TruHTAmp7Rv	5'-CAAGCAGAAGACGGCATACGAGA-3'	Strand synthesis
TruHTAmp5Fw	5'-AATGATACGGCGACCACCGAGATCTAC-3'	Library PCR
TruHTAmp701allSRv	5'-CA*A*G*C*A*G*A*A*G*A*C*G*G*C*A*T*A*C*G*A*G*A*T*C *G*A*G*T*A*A*T-3'	Library PCR

* Phosphorothioate bond.

Doc. S1

Supplemental methods

Yeast DNA preparation

Yeast pellets were suspended in 500 μl of 0.2 M Tris-Cl pH8.5 with 25 μl of β-mercaptoethanol and incubated at 30°C for 30 min. After removing the buffer, cells were resuspended in 40 mM Sodium phosphate pH6.8, 1 M Sorbitol with 1.5 mg/ml of Zymolyase 100T (Nacalai) and icubated at 37°C for 1 h. Recovered cells were lysed in pre-warmed lysis buffer (50 mM Tris-Cl pH7.5, 0.2 M NaCl, 0.1 M EDTA, 5% SDS) at 65°C by gentle pipetting and inversions. After phenol-chloroform extraction twice, nucleic acids were precipitated with twice volume of 99% ethanol, and the precipitates were rinsed with 70% ethanol. Dried nucleic acids were treated by RNase A (Wako) in 500 μl of RNase buffer (10 mM Tris-Cl pH7.5, 1 mM EDTA, 0.5 mg/ml RNase A) at 37°C for 2 h. After phenol-chloroform extraction twice, genomic DNAs were precipitated with twice volume of 99% ethanol, and the precipitates were rinsed with 70% ethanol. Pellets were dissolved in 2.5 mM Tris-Cl pH 7.5 and stored at 4°C. Note, all pH is at 25°C. Genomic DNA concentration was measured by Qubit2.0 Fluorometer with dsDNA HS assay kits (Thermofisher).

Standard DNA preparation

Standard DNAs (STDs) were synthesized from ΦX174RFI DNA (NEB, SD0031, Lot. 00121773) by PCR and rNMP adaptor ligation. Background fragments were amplified by PCR with KOD FX plus polymerase (TOYOBO), primer sets (STD1: STDf1Fw-STDf1Rv and STD2: STDf2Fw-STDf2Rv) and thermal-cycle condition [94°C, 1 min, (98°C, 7 sec., 50°C, 15 sec., 68°C, 40sec.) x 30 cycles, 4°C]. After gel purification, the STD fragments were digested by StyI-HF (NEB) at 37°C overnight. Gel-purified STD/StyI fragments were ligated with rNMP adaptor sets (rAFw-rGRv and rCFw-rURv) in 100 μl reaction (1xCutSmart buffer (NEB), 0.2 mM ATP, 2 μg STD, 50 U StyI-HF, 50 U XbaI (NEB), $1x10^{4}$ U T4 DNA-ligase (NEB), 5 μM rNMP adaptors) at 20°C for 3 days. Ligation products were gel purified as STD-rNMP fragments. For adaptor preparation, each 100 μM primer set (STDrA-STDrA_Rv, STDrG-STDrG_Rv, STDrC-STDrC_Rv or STDrU-STDrU_Rv) in annealing buffer (10 mM Tris-Cl pH8.0, 50 mM NaCl) was heat-denatured at 95°C for 5 min and annealed by gradual lowering to room temperature.

Alkali-denaturing agarose gel analysis

Alkaline digestion and alkaline agarose electrophoresis were performed with 2 μg of genomic DNA on 0.8% agarose gels as previously described (Nick McElhinny et al., 2010). After electrophoresis, gels were stained with GelRed (Biotium) for 10 hours, and images were captured on a FUSION SL system (Vilber-Lourmat) equipped with a UV trans-illuminator. The mean length of alkali-digested DNAs was quantified using the Image Quant TL software (GE Healthcare).

For Southern analysis of RNase H–treated DNAs, 4 μg of genomic DNAs was digested with FastDigest

(FD)-*Dra*I (Chr. III) or FD-*Bgl*II (Chr. XII) (Fermentas) at 37°C overnight. After purification of restriction fragments using AMPure XP beads (Beckman Coulter) (DNA solution : AMPure, 1:1.5), restriction DNA fragments and 100 ng of PCR fragments (Chr. III: 206,367–214,895 and Chr. XII: 848,199–855,899) were treated with RNase H (20 U) and HII (20 U) in 40 μl of 1× ThermoPol reaction buffer (NEB) at 37°C overnight. After AMPure XP bead purification (1:1.5), RNase H–treated DNAs were separated on alkaline agarose gels as described above and capillary-transferred to Hybond N^{+} membranes prior to depurination (0.1 N, HCl for 20 min) and neutralization [1 M Tris-Cl (pH 8.1), 1.5 M NaCl for 30 min]. Southern hybridization was performed using ^{32}P-α-dCTP random-primed labeled PCR fragments as probes (Chr. III: 206,367-207,369 and Chr. XII: 848,199-849,112). Southern blot images were captured on a FLA-7000 (GE Healthcare), and lane profiles were analyzed using Image Quant TL. In Figure S2E and S2F, the input level of Southern blot image was adjusted to 25% in a raw tiff file by Adobe Photoshop CS6.

RiSQ-seq protocol (library preparation)

Solution:

- microTUBE: microTUBE Snap-Cap AFA Fiber (Covaris)
- AMpureXP beads (Beckman Coulter)
- Elution buffer: 5 mM Tris-acetate pH8.0.
- 10x End-repair buffer: 500 mM Tris-HCl pH7.5, 100 mM MgCl2 100 mM DTT.
- End-repair enzyme: End-repair enzyme mix from KAPA kit*.
- 10 mM ATP
- 2.5 mM dNTP mix (Takara)
- SeaKem GTG agarose (Lonza)
- Micro Bio-Spin column (BIO-RAD)
- 1x TAE: 40 mM Tris, 20 mM acetic acid, 1 mM EDTA
- STD mix: standard DNA mix (See "Standard DNA preparation" section)
- *KAPA library preparation kit for illumina (KAPA biosystems)
- 30 μM TruHT adaptor: 3'-end masked illumina TruSeqHT compatible adaptor from primer set annealing (TruHTAdaptor702TddC with TruHTAdaptor501IddT, TruHTAdaptor502IddT, TruHTAdaptor503IddT or TruHTAdaptor504IddT).
- 5x TDT buffer: 0.5 M HEPES pH7.2, 40 mM $MgCl_2$, 5 mM $CoCl_2$, 0.5 mM DTT, 0.1% BSA.
- 2.5 mM ddNTP mix (Roche)
- Terminal deoxynucleotidyl transferase (TDT) (Takara)
- 0.5 M EDTA (pH8.0)
- 2xAMpureXP: AMpureXP beads are resuspended in half volume of 2.5M NaCl, 20% PEG 8,000.
- 10x ThermoPol buffer (NEB)

- RNase HI: RNase H (NEB)
- RNase HII (NEB)
- 5x Ligation buffer: 330 mM Tris-HCl pH7.6, 50 mM $MgCl_2$, 5 mM DTT, 30% PEG 6,000.
- T4 DNA ligase: T4 DNA ligase from from KAPA kit*.
- 100 μM ssLig adaptor: secondary adaptor from primer set annealing (TruHTAdaptor701_ss and ssLigAdaptor-i7short).
- Biding support buffer: 1M NaCl, 20mM MgCl2, 20mM TrisHCl pH7.8.
- BstPol mix: 1xThermoPol buffer, 3.2 U/μl Bst DNA Polymerase, Large Fragment (NEB).
- KAPA real-time library amplification kit (KAPA biosystems)
- Primers: see Table S2.

Protocol:

Genomic DNA (gDNA) fragment preparation

1. Genomic DNA (10 μg in 100 μl) of was shared by CovarisS220 (Covaris) in microTUBE at 4°C, peak power 140 W, duty factor 10%, cycle per burst 200 and duration time 1 minute.
2. Purify DNA fragments by mixing with AMpureXP beads (DNA : AMpureXP = 1:1), incubation at room temperature for 15 min, beads recovery, 80% ethanol beads rinse twice, vacuum dry for 5 min and elution with 30 μl elution buffer.
3. End-repair DNA fragments in 50 μl reaction (1x End-repair buffer, 2 mM ATP, 0.5 mM dNTP, 2.5 μl End-repair enzyme) at 20°C for 30 min.
4. Gel-purify 400-450bp length DNAs by 1.5% SeaKem GTG agarose gel electrophoresis (1xTAE), ethidium bromide staining and freeze-squeezing DNA recovery*. * Freeze-squeezing DNA recovery: Gel slices are frozen at -80°C for 5 min, and recover the DNA solution by centrifugation through Micro Bio-Spin column at room temperature. Recovered DNA is purified by AMpureXP beads (1:1).
5. Elute DNA fragments (gDNA) in 50 μl of elution buffer.

Primary library preparation and 3'-end masking

6. Mix 1 μg of gDNA and 25 ng of STD mix for primary library preparation with KAPA library preparation kit for illumina and 30 μM TruHT adaptor (501-702 set for gDNA libraries).
7. Prepare gDNA library according to the manufacturer's protocol.
8. Elute gDNA library in 15.5 μl of elution buffer.
9. Mask free 3'-hydroxy group of gDNA library by TDT in 22 μl reaction (1x TDT buffer, 0.25 mM ddNTP, 10 U TDT, 14 μl gDNA library) at 37°C for 1h.
10. Stop TDT reaction by adding 3 μl of 0.5 M EDTA.
11. Purify 3'-end masked library by 2xAMpureXP (1:1) with 12.5 μl elution.
12. Gel-purify fully adaptor-ligated library around 1.5kbp size** by 2% SeaKem GTG agarose gel electrophoresis (1xTAE), SYBR-Green staining and freeze-squeezing DNA recovery. ** Fully adaptor-

ligated library migrates slower than estimated length by branching adaptors.

13. Elute primary library in12.5 μl of elution buffer.

RNase H treatment of primary library

14. Digest primary library by RNase HI and HII in 15 μl reaction (1x ThermoPol buffer, 3.75 U RNase HI, 3.75 U RNase HII, 100 ng primary library) at 37°C for 18 h.
15. Purify RNase H treated library by 2xAMpureXP (1:1) with 5 μl elution.
16. Check library concentration.
17. Heat-denature 30 ng of RNase H treated library at 98°C for 3 min and chill at 4°C for 5 min.

Secondary adaptor ligation

18. Ligate secondary adaptor to denatured library in 12.5 μl reaction (1x Ligation buffer, 1 mM ATP, 10 μM ssLig adaptor, 1.25 μl T4 DNA ligase, 30 ng denatured library†) at 20°C for 12 h. † For library without RNase H treatment, 30 ng of library at step 13 are denatured as in step 17.
19. Add 12.5 μl of Binding support buffer.
20. Purify by 2xAMpureXP (1:0.8) with 12.5 μl elution.
21. Repeat step 19-20.
22. Add 12.5 μl of Binding support buffer.
23. Purify single-stranded library by 2xAMpureXP (1:0.8) with 10 μl elution.

Complementary strand synthesis

24. Anneal primer and single-stranded library in 20 μl reaction (1x ThermoPol buffer, 0.32 mM dNTP, 5 μM TruHTAmp7Rv, 10 μl single-stranded library) by heat-denaturation and annealing (95°C 5min, 57°C hold).
25. Add 5 μl of BstPol mix at 57°C and synthesis complementary strand (57-65°C 10 min, 65°C 10 min, 80°C 20 min and 4°C hold).
26. Purify single-stranded library by 2xAMpureXP (1:1) with 6 μl elution.
27. Load into illumina sequencer as PCR-free library.

Library amplification by PCR

28. Amplify rNMP library by KAPA real-time library amplification kit with a primer set (TruHTAmp5Fw and TruHTAmp701allSRv) in 50 μl reaction (1x Realtime RM mix, 1μM primer set, 5 ng library step 26) under thermal cycle condition [98°C, 45 sec, (98°C, 15 sec., 58°C, 30 sec., 72°C, 35sec.) x 10-22 cycles].
29. Purify single-stranded library by 2xAMpureXP (1:0.8) with 25 μl elution.
30. Add 25 μl of Binding support buffer.
31. Purify single-stranded library by 2xAMpureXP (1:0.8) with 12.5 μl elution.
32. Load into illumina sequencer as PCR-amplified library.

Data processing

Data quality control

All the RNase H treated samples and/or YPD cultured samples were replicated. Sequencing quality control was checked by Illumina Sequencing Analysis Viewer 1.8.20 (illumina) at cluster density (<1,500 K/mm^2), cluster pass-filter (>80%) and total Q30 (>80%). Read quality control was checked by FastQC 1.0 (http://www.bioinformatics.babraham.ac.uk/projects/fastqc). Mapping data quality was checked by Qualimap 2.1 (García-Alcalde et al., 2012).

Sequence read mapping and coverage counting

Reference sequences of *S. cerevisiae* sequences for read mapping were from the ensemble build (EF4.68) of the S288C reference genome, and chromosome numbering was changed from Roman numerals to Arabic numerals. For Ty1 retrotransposon analysis in the S288C genome, 31 Ty1 retrotransposons with 1 kb franking sequences were used for mapping. Ty1 consensus sequence was from CLUSTALW alignments (Larkin et al., 2007) of the 31 Ty1 sequences. For mapping to rDNA repeat unit sequence (chr12:458,433-467,569) and mitochondrial genome sequence (chrmt:1-85,779), rDNA and chrmt sequences were shift to 5 kb and 10 kb downstream, respectively, to compensate mapping bias around sequence ends, and both original- and shifted-references were used for mapping. In STD fragment analysis, ΦX174 based synthetic fragment sequences with or without SNPs were used as reference sequences.

Read mapping was performed by bowtie 1.0.0 (Langmead, Trapnell, Pop, & Salzberg, 2009) onto reference sequences: paired-end read mapping (-S --fr -k 1 -n 3 -X 1000) for genomic reads, and single-read mapping of read2 set (-S --best -k 1 -n 0) for STD reads. After selecting properly mapped pairs by SAMtools 0.1.19 (Li et al., 2009) , the each pair of genomic reads in SAM files was directly converted to single forward (read1 direction) strand fragment (both background and rNMP reads) and rNMP position (immediately 3' of rNMP reads) in BED files by AWK scripts. SAM data of STD reads were directly converted to read count data of each STD fragments with SNPs and mapped-end position by AWK scripts. Coverage of mapped fragments and rNMPs onto single-base bin files in BED format was computed by bedtools 2.24.0 (Quinlan & Hall, 2010) (coverageBed -s). These coverage data were mainly used for rNMP accumulation analysis in following processes.

STD fragment analysis

Input rNMP rates were estimated from primary library sequencing by SNP detection of STD fragments:

$$rXMP_{\text{input rate (X=A,C,G,U)}} = rXMP_{\text{fragment}} / (rNMP_{\text{fragment (N=A\&C or G\&U)}} + STD_{\text{background}})$$

RiSQ-seq rNMP rates were estimated from read counts mapped to proper ends of STD fragments:

$$rXMP_{\text{RiSQ-seq rate (X=A,C,G,U)}} = rXMP_{\text{rXMP-end}} / (rNMP_{\text{rNMP-end (N=A\&C or G\&U)}} + rNMP_{\text{full-end}} + STD_{\text{full-end}})$$

Recovery rates were calculated by normalization of RiSQ-seq rNMP rate with rNMP rate:

$$rXMP_{\text{recovery rate (X=A,C,G,U)}} = rXMP_{\text{RiSQ-seq rate}} / rXMP_{\text{input rate}}$$

rNMP recovery rates were mean average of $rXMP_{\text{(X=A,C,G,U)}}$. For rNMP adjustment, a reciprocal of rNMP detection efficiency, R_{standard} was estimated by linear regression model by R ver. 3.2.1 (http://www.R-project.org/) function [lm($rXMP_{\text{input rate (X=A,C,G,U)}} \sim rXMP_{\text{RiSQ-seq rate}}$ +0)].

To adjust RNase H untreated data, ligation efficiency ($R_{\text{standard mock}}$) was estimated from RNase H digestion efficiency (RDG) and rNMP detection efficiency in RNase H treated sample. Firstly, rNMP fragments without

RNase H digestion in background reads were normalized by total STD background reads as undigested rates. Next, RNase H digestion rates (rXMP $_{\text{digestion rate (X=A,C,G,U)}}$) were estimated by subtraction of undigested rates from input rNMP (rXMP $_{\text{input rate}}$) rates. RNase H digestion efficiency, RDG was estimated by linear regression model by R [lm(rXMP $_{\text{input rate (X=A,C,G,U)}}$ ~ rXMP $_{\text{digestion rate}}$ +0)]. Finally, $R_{\text{standard mock}}$ was calculated by normalization of R_{standard} by RDG: $R_{\text{standard mock}} = R_{\text{standard}}$ / RDG

PCR library adjustment

For scale correction of PCR-amplified rNMP data, rNMP coverage onto full-length chromosome bins was computed and analyzed by linear regression analysis without intercept. R_{pcr} was estimated by R function: lm(PCR_rNMP$_{\text{chr.strands}}$~ PCR-free_rNMP$_{\text{chr.strands}}$ +0). For control data, immediately 3' positions of all properly mapped fragments (total background end: Bg-end) in RNase H untreated samples were adjusted to scale of secondary-adaptor reads (SA-end) as random targets of secondary adaptor ligation. Correction coefficients for control data R_{control} were also estimated by R function: lm(Bg-end$_{\text{chr.strands}}$ ~ SA-end$_{\text{chr.strands}}$ +0).

Estimation of rNMP accumulation

rNMP accumulation was estimated from read coverage of rNMP and background at single base bins on each strand. Except a single base meta analysis of mitochondrial rNMP (Figure S4F), all rNMP accumulation values were analyzed with 42- (whole genome) or 21-base (Ty1 and rDNA units) bins that are consist of 7 trios of base blocks and sliding with 14 or 7 base step (see below). Mean value of the 7 trios was accounted to be rNMP accumulation level at the center 14 or 7 base bin. These trios in each bin allowed providing non-overlapped data sets and comparing different samples.

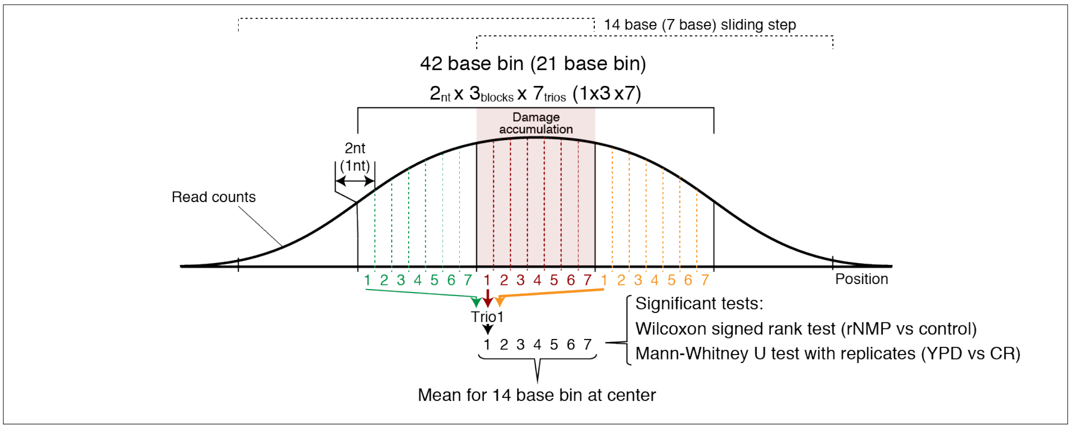

rNMP accumulation in each trio ($\text{rNMP}_{\text{rate i}}$) was calculated from total rNMP count in PCR library (rNMP_{PCR}), total background count in PCR-free library (Bg), PCR-correction coefficient (R_{pcr}) and standard adjustment coefficient (R_{standard}):

$$\text{rNMP}_{\text{rate i}}\,(\text{dpm}) = \frac{\text{rNMP}_{\text{PCR}}}{R_{\text{PCR}} \cdot \text{Bg} + \text{rNMP}_{\text{PCR}}} \cdot R_{\text{standard}} \cdot 10^6$$

For mitochondrial rNMP accumulation, $\text{rNMP}_{\text{rate i}}$ was calculated from total rNMP count in PCR-free library (rNMP), total background count in PCR-free library (Bg) and standard adjustment coefficient (R_{standard}):

$$\text{rNMP}_{\text{rate i}}\,(\text{dpm}) = \frac{\text{rNMP}}{\text{Bg} + \text{rNMP}} \cdot R_{\text{standard}} \cdot 10^6$$

The $rNMP_{rate}$ accumulation at 42- or 21-base bin (rNMP) was mean value of $rNMP_{rate\ i}$ at 7 trios:

$$rNMP_{rate}\ (dpm) = \frac{\sum_{i=1}^{7} rNMP_{rate\ i}}{7}$$

The control level was estimated from background count (Bg_{mock}), background 3' end count ($Bg_{mock\ end}$)in RNase H- and PCR-free library, correction coefficients for control data $R_{control}$ and ligation efficiency ($R_{standard\ mock}$):

$$rNMP_{control\ i}\ (dpm) = \frac{Bg_{mock\ end}}{R_{control} \cdot Bg_{mock} + Bg_{mock\ end}} \cdot R_{standard\ mock} \cdot 10^6$$

$$rNMP_{control}\ (dpm) = \frac{\sum_{i=1}^{7} rNMP_{control\ i}}{7}$$

Extraction of biased regions

Since multiple copy sequences tended to show biased coverage of background reads, this study excluded such regions from rNMP accumulation analysis. Precise profiles of rDNA repeat unit and Ty1 retrotransposons were separately analyzed at 21 base bin level. To extract biased region, background reads (YPD, background reads in WT and *rnh201Δ* samples) were mapped onto reference sequences by paired-end and single-end read modes by bowtie 1.0.0 (paired-end mode: --fr -S -k 1 -n 3 -X 1000, single-end mode: -k 1 -S -n 3 --best). After conversion of mapping data to coverage data of 252 base bins with 14 base sliding step by SAMtools and bedtools, regions in which coverage rate between paired- and single-end mapping was over 1.5 were selected from both Watson and Crick strand and merged by bedtools (mergeBed). Biased clusters over 1 kb, telomere proximal regions (0.5 kb from telomere end), rDNA region and 5S rDNAs were combined as biased regions.

Read distribution analysis

Using PCR-amplified rNMP coverage data on single base bins, total rNMP counts on nuclear genome without biased regions were calculated for normalization. $rNMP_{PCR}$ coverage data on 42-base bins with 14-base sliding step were normalized by total rNMP counts and bin size of each sample (reads per million / base: rpm / base).

GC content estimation and peak selection

GC content was calculated from 126 base window sliding with 14 base step (bed file) that associated with 14 base bin at the center of the window by bedtools and reference sequences fasta (nucbed -fi reference.fasta -bed 126-base_window.bed). GC content of genes, nucleosomes, and other segments were calculated from mean GC content of associated bins. GC- (GC>0.5) and AT-peaks (GC<0.2) were selected as peak center bins in successive 5 windows with enough length of intervals: the interval of GC-peaks was over 250 bases, and 500 bases for AT-peaks. The replication direction of each peak was classified by Okazaki fragment rate described below.

Parameter for replication direction

For evaluate rate of replication direction at each genomic region, fragment coverage of Okazaki fragment sequencing data in previously study was used (Smith & Whitehouse, 2012) Paired-end reads data (WT_sample data: SRR364781) were mapped onto reference sequences by bowtie 1.0.0 (-S --fr -k 1 -n 3 -X 1000), the resultant mapping data (SAM) were converted to fragment data (BED), and coverage of Okazaki fragments

(OF) at single-base bins of Watson and Crick strands were computed by bedtools (-s coverageBed). Total coverage of OF at 238 base windows on Watson (OF_{Watson}) and Crick (OF_{Crick}) strands were converted to OF rate (OF_{rate}) data for 14 base bins at the center of the windows:

$$OF_{rate} = \frac{OF_{Watson}}{(OF_{Watson} + OF_{Crick})}$$

$OF_{rate}>0.5$ indicates left to right replication major and $OF_{rate}<0.5$ indicates right to left replication.

OF_{bias} was also used for selection of unidirectional replicated regions:

$$OF_{bias} = \max(OF_{rate}, 1 - OF_{rate})$$

The OF_{rate} of genes, nucleosomes, and other segments were calculated from mean OF_{rate} of associated bins.

Simple repeats calling

Homo-polymers, dinucleotide and triplet repeats in *S. cerevisiae* genome were searched by non-B DB v2.0 (Cer et al., 2013) and classified by repeat types for analysis.

Spontaneous mutations in S288C genome

Previously reported mutations (GSE56939_variants.txt) were converted from original sequences to S288C reference sequences by blast-2.2.26, and properly mapped mutations (indels: N=1012, base substitutions: N=294) were used for analysis (Lujan et al., 2014).

DIPs among *S. cereviae* strains

To analyze natural DIPs among 37 *S. cerevisiae* strains, alignment data were compressed to variation counts at each position (ftp://ftp.sanger.ac.uk/pub/users/dmc/yeast/latest/misc.tgz, /misc/multipleSpeciesAlignments /chrXX/sequenced.gz, sequenced, XX: Arabic numeral of chromosome number.) (Liti et al., 2009). After extraction of DIP counts, S288C EF1 data were converted to EF4 reference data by Ensemble Assembly Converter. DIP rates at 42-base bins were calculated from EF4 converted data.

RTM peaks collection

Peaks and valleys of RTM were collected from RTM lists as maximums and minimums in 5 successive 42-base bins.

ROC curve analysis

ROC curve analysis was performed by R package ROCR (Sing, Sander, Beerenwinkel, & Lengauer, 2005). RMT thresholds of accuracy over 0.99 (False positive rate <0.01) were estimated from threshold lists. Regions over 0.99 accuracy thresholds were extracted from RTM lists with 14 base bins (BED) and merged them by bedtools (mergeBed).

References for Supplemental Methods

Cer, R. Z., Donohue, D. E., Mudunuri, U. S., Temiz, N. A., Loss, M. A., Starner, N. J., … Stephens, R. M. (2013). Non-B DB v2.0: A database of predicted non-B DNA-forming motifs and its associated tools. *Nucleic Acids Research*, *41*(Database issue), D94–D100. https://doi.org/10.1093/nar/gks955

García-Alcalde, F., Okonechnikov, K., Carbonell, J., Cruz, L. M., Götz, S., Tarazona, S., … Conesa, A. (2012). Qualimap: Evaluating next-generation sequencing alignment data. *Bioinformatics (Oxford, England)*,

28(20), 2678–2679. https://doi.org/10.1093/bioinformatics/bts503

Langmead, B., Trapnell, C., Pop, M., & Salzberg, S. L. (2009). Ultrafast and memory-efficient alignment of short DNA sequences to the human genome. *Genome Biology*, *10*(3), R25. https://doi.org/10.1186/gb-2009-10-3-r25

Larkin, M. A., Blackshields, G., Brown, N. P., Chenna, R., McGettigan, P. A., McWilliam, H., … Higgins, D. G. (2007). Clustal W and Clustal X version 2.0. *Bioinformatics (Oxford, England)*, *23*(21), 2947–2948. https://doi.org/10.1093/bioinformatics/btm404

Li, H., Handsaker, B., Wysoker, A., Fennell, T., Ruan, J., Homer, N., … 1000 Genome Project Data Processing Subgroup. (2009). The Sequence Alignment/Map format and SAMtools. *Bioinformatics (Oxford, England)*, *25*(16), 2078–2079. https://doi.org/10.1093/bioinformatics/btp352

Lujan, S. A., Clausen, A. R., Clark, A. B., MacAlpine, H. K., MacAlpine, D. M., Malc, E. P., … Kunkel, T. A. (2014). Heterogeneous polymerase fidelity and mismatch repair bias genome variation and composition. *Genome Research*, *24*(11), 1751–1764. https://doi.org/10.1101/gr.178335.114

Nick McElhinny, S. A., Watts, B. E., Kumar, D., Watt, D. L., Lundström, E.-B., Burgers, P. M. J., … Kunkel, T. A. (2010). Abundant ribonucleotide incorporation into DNA by yeast replicative polymerases. *Proceedings of the National Academy of Sciences of the United States of America*, *107*(11), 4949–4954. https://doi.org/10.1073/pnas.0914857107

Quinlan, A. R., & Hall, I. M. (2010). BEDTools: A flexible suite of utilities for comparing genomic features. *Bioinformatics (Oxford, England)*, *26*(6), 841–842. https://doi.org/10.1093/bioinformatics/btq033

Sing, T., Sander, O., Beerenwinkel, N., & Lengauer, T. (2005). ROCR: Visualizing classifier performance in R. *Bioinformatics (Oxford, England)*, *21*(20), 3940–3941. https://doi.org/10.1093/bioinformatics/bti623

Smith, D. J., & Whitehouse, I. (2012). Intrinsic coupling of lagging-strand synthesis to chromatin assembly. *Nature*, *483*(7390), 434–438. https://doi.org/10.1038/nature10895
